# BIGwas: Single-command quality control and association testing for multi-cohort and biobank-scale GWAS/PheWAS data

**DOI:** 10.1093/gigascience/giab047

**Published:** 2021-06-29

**Authors:** Jan Christian Kässens, Lars Wienbrandt, David Ellinghaus

**Affiliations:** Institute of Clinical Molecular Biology, Christian-Albrechts-University of Kiel, Rosalind-Franklin-Str. 12, 24105 Kiel, Germany; Haematology Lab Kiel, Klinik für Innere Medizin II, University Hospital Schleswig-Holstein, Langer Segen 8-10, 24105 Kiel, Germany; Institute of Clinical Molecular Biology, Christian-Albrechts-University of Kiel, Rosalind-Franklin-Str. 12, 24105 Kiel, Germany; Institute of Clinical Molecular Biology, Christian-Albrechts-University of Kiel, Rosalind-Franklin-Str. 12, 24105 Kiel, Germany; Novo Nordisk Foundation Center for Protein Research, Disease Systems Biology, Faculty of Health and Medical Sciences, University of Copenhagen, Blegdamsvej 3b, 2200 Copenhagen, Denmark

**Keywords:** GWAS, PheWAS, biobank, quality control, association testing, pipeline, Nextflow, Singularity, scalability

## Abstract

**Background:**

Genome-wide association studies (GWAS) and phenome-wide association studies (PheWAS) involving 1 million GWAS samples from dozens of population-based biobanks present a considerable computational challenge and are carried out by large scientific groups under great expenditure of time and personnel. Automating these processes requires highly efficient and scalable methods and software, but so far there is no workflow solution to easily process 1 million GWAS samples.

**Results:**

Here we present BIGwas, a portable, fully automated quality control and association testing pipeline for large-scale binary and quantitative trait GWAS data provided by biobank resources. By using Nextflow workflow and Singularity software container technology, BIGwas performs resource-efficient and reproducible analyses on a local computer or any high-performance compute (HPC) system with just 1 command, with no need to manually install a software execution environment or various software packages. For a single-command GWAS analysis with 974,818 individuals and 92 million genetic markers, BIGwas takes ∼16 days on a small HPC system with only 7 compute nodes to perform a complete GWAS QC and association analysis protocol. Our dynamic parallelization approach enables shorter runtimes for large HPCs.

**Conclusions:**

Researchers without extensive bioinformatics knowledge and with few computer resources can use BIGwas to perform multi-cohort GWAS with 1 million GWAS samples and, if desired, use it to build their own (genome-wide) PheWAS resource. BIGwas is freely available for download from http://github.com/ikmb/gwas-qc and http://github.com/ikmb/gwas-assoc.

## Introduction

Genome-wide association studies (GWAS), in which millions of genetic variants are tested across the genomes of study individuals to identify genotype-phenotype associations, have revolutionized the field of complex disease genetics over the past 10 years. Decreasing costs of genome-wide genotyping with single-nucleotide polymorphism (SNP) arrays allow routine collection of GWAS data from deeply phenotyped biobank population cohorts, where many clinical features can be assessed beyond disease status. For this reason, it is possible to conduct GWAS studies with >1 million samples [1] using GWAS data from large biobanks such as the UK Biobank (UKB) [2] or the US Million Veterans Program [3]. This further allows phenome-wide association studies (PheWAS) to be conducted to systematically study the effects of 1 or many genetic variants over a wide range of human phenotypes. GWAS/PheWAS analyses with >1 million GWAS samples from dozens of biobanks enable, for example, rapid screening for target genes for therapeutic interventions for COVID-19 patients in the current pandemic [4]. However, while efficient software pipelines, such as PheWeb [5], for visualization, navigation, and sharing of results of large-scale GWAS and PheWAS projects have been developed recently, there are still no easily executable and reproducible software pipelines available for performing large-scale quality control (QC) and association tests on a large number of individual (large-scale) GWAS or PheWAS data sets with 1 million GWAS samples. In particular, parallel and consistent processing and analysis of dozens of individual GWAS cohorts in the context of a mega-GWAS analysis is enormously time-consuming and can quickly become unmanageable if processed manually.

For basic GWAS QC and association analysis, software programs such as PLINK2 [6] can efficiently process and analyse whole-genome genotype data. Owing to its broad functionality and efficient binary file format, PLINK2 is commonly used, along with self-written R and Bash scripts for visualization and further processing of files and results. To facilitate the execution and chaining of numerous PLINK commands and R programs, several data-processing packages for GWAS analysis have been developed in R [7,8] or other scripting languages [9]. In addition, software environments such as RICOPILI [10] or Odyssey [11] have been developed with advanced online manuals that bundle many Unix, Awk, Perl, and/or PLINK2 commands for QC and association analysis. However, familiarization with these environments is usually time-consuming and is complicated by the fact that the user must perform a large number of installation steps for their own working environment and/or modify and manually execute a collection of Bash and R scripts in specific directories. Odyssey, for example, supports the use of software containers but lacks high-performance compute (HPC) support and thus the scalability for large GWAS data sets. Recently, the portable and reproducible GWAS QC and association testing software pipeline H3Agwas (currently version 3) has been developed by Baichoo and colleagues from the Pan-African community project H3ABioNet [12], which takes the input and output data from various QC and association testing automatically through several computationally intensive processing steps. These workflows have been generated for processing small GWAS data sets but were not recommended for processing data sets with >50,000 GWAS samples. To our knowledge, there is no portable (i.e., container-based) QC and association software pipeline that (i) can be executed with a single command without manual pre-installation, (ii) does not require reading and executing long online tutorials, and (iii) can efficiently and quickly process 1 million GWAS samples as provided by large-scale GWAS/PheWAS biobank projects.

**Figure 1:**
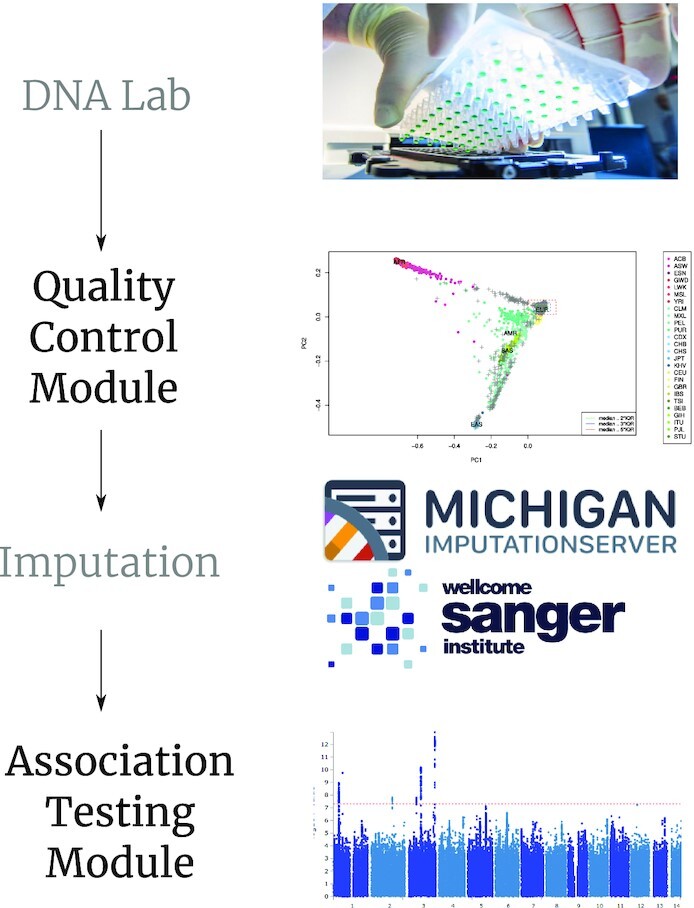
Overview of the single-command quality control and association testing workflow of BIGwas. Multiple GWAS data sets in PLINK format from any genotyping platform can be used as input for the Quality Control (QC) Module. Both the QC Module and the Association Testing Module can be started and executed with a single command. The genotype output files of QC Module in VCF format can be used directly as input for genotype imputation programs such as the TOPMed Imputation Server [20]. The VCF output files of the imputation process can be used directly as input for the Association Testing Module, which implements logistic/linear regression and mixed model association testing using PLINK [6] and SAIGE [21].

We developed BIGwas, a fast and efficient platform-independent software pipeline for QC and association testing of large-scale GWAS/PheWAS data sets, which automatically processes 1 million GWAS samples. By using Nextflow workflow technology  [13] in combination with Singularity software container technology [14] (Methods), BIGwas can be started with a single command (Fig. 1), with no need to manually install a full software execution environment or various software packages. BIGwas implements a variety of best-practice QC and GWAS mega-analysis protocols (see Methods) developed by the Inflammatory Bowel Disease Genetics Consortium [15–17] and the Severe Covid-19 GWAS Group [18]. The development of our container-based and reproducible biobank-scale GWAS/PheWAS software pipeline was further motivated by current large-scale GWAS and PheWAS screening projects, such as the UKB GWAS/PheWAS project of Benjamin Neale and colleagues (http://www.nealelab.is/uk-biobank/) or the Michigan Genomics PheWeb Initiative (http://pheweb.sph.umich.edu/about) in which GWAS analyses are performed for thousands of human traits with large GWAS data sets from UKB and the Michigan Genomics Initiative. The current PheWeb instance of the Michigan Genomics Initiative displays UKB summary statistics of 28 million genetic markers assessed across 1,403 binary traits for 408,961 white British participants [19]. In these projects, the underlying QC and GWAS/PheWAS association analyses for thousands of traits run on large HPCs for several months or even years and are performed by large scientific working groups under great expenditure of time and personnel. To create a (genome-wide) PheWAS resource of this type, BIGwas can be used alongside the QC and mega-GWAS analysis capability to test for association with a wide variety of traits in parallel or sequentially (requiring only 1 single program call per trait on an HPC) when numerous different phenotype information is available for the same individuals.

BIGwas comprises 2 different modules, namely, the Quality Control (QC) Module and the Association Testing Module, that are part of a typical GWAS workflow (Fig. 1). While the QC module uses user-supplied GWAS data sets in the widely adopted PLINK binary format, the Association Testing Module also allows input formats such as VCF (variant call format) that are usually delivered by genotype imputation servers, as with the TOPMed Imputation Server [20]. We compared the performance of BIGwas with the H3Agwas Pipeline Version 3 in terms of runtime and functionality because H3Agwas is currently the only portable pipeline for reproducible GWAS that can be installed from a single source and started with just a few commands. To perform the benchmarks, we used both small (≤20,000 GWAS samples) as well as large GWAS data sets (≤1 million GWAS samples), including the current genotyped and imputed GWAS releases of UKB (September 2020) for COVID-19 case-control association analysis.

## Methods

### Quality control module

**Figure 2:**
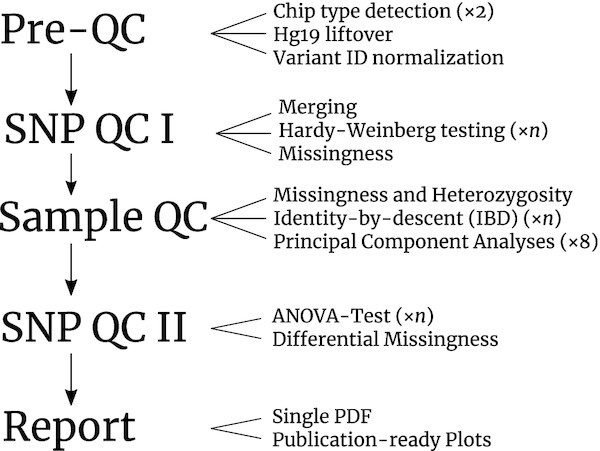
Workflow graph of the Quality Control (QC) Module of BIGwas, with the default number of threads used in each process (for details see Methods). *n* denotes that the number of parallel jobs or threads automatically scales with the number of samples in the data set.

The QC module works for binary as well as quantitative traits and is subdivided into 5 distinct phases: Pre-QC, SNP QC I, Sample QC, SNP QC II, and Final Analysis and Report Generation. For a QC based on quantitative traits, in the PLINK input Fam file, only the sixth column must be specified as a quantitative trait. In Pre-QC, an unlimited number of input GWAS data sets (batches) in PLINK format from different Affymetrix and Illumina platforms are, among other steps, matched with known variant databases from dbSNP and currently 140 Illumina/Affymetrix GWAS platforms and then brought to the same marker content. SNP QC I primarily filters variants on the basis of missingness and Hardy-Weinberg equilibrium (HWE; in controls only) across and within batches (i.e., genotype files or self-defined batches) as well as batches from a particular phenotype and the entire control group with a user-defined false discovery rate (FDR) threshold, allowing for differences in sample size between batches. The Sample QC removes samples based, among others, on missingness, increased or decreased heterozygosity rates, duplicate/relatedness testing (identity-by-state [IBS] and identity-by-descent [IBD] estimation), and population structure analysis/Tracy-Widom statistics, with or without projection on reference samples from HapMap and the 1000 Genomes Project. SNP QC II filtering steps include testing for differential missingness between phenotypes and controls, monomorphic sites, and significantly different allele frequencies of variants across the batches from a particular phenotype or the control group while correcting for population structure or ancestry via analysis of variance (ANOVA), with a user-defined FDR threshold. Finally, all information gathered throughout the QC pipeline is written to a single PDF report (see Fig. 2). In principle, the QC module can be used for any European and non-European population. However, for the special case of an intended trans-ancestry GWAS study (e.g., to analyse GWAS samples of European, Asian, and African descent), we recommend that a separate QC be performed for each ethnic group.

### Pre-QC

In large-scale consortium-based GWAS with dozens of GWAS input data sets collected from different gentoyping centers it is useful to apply SNP and sample QC metrics across and within genotyping batches for the identification of potential batch effects. For this task and the subsequent merging of data sets from the same genotyping platforms, all genetic variants are annotated in the same way. This includes the mapping to a genome build, the naming of variants as provided by the dbSNP databases, and the strand alignment. The Pre-QC phase brings all input data sets to a common annotation that is suitable for merging.

#### Individual annotations

A batch affiliation can be defined independently from a single GWAS batch using the annotations file. In this file, separate batch identifiers can be assigned for the genotyping batch, country of origin, and diagnosis (if non-control). Each of these identifiers will be used in principal component (PC) plots, histograms, and ANOVA tests to identify batch effects within input data sets.

#### Assignment of variants to pseudo-autosomal regions

The first step consists of checking and assigning variants to the pseudo-autosomal regions (PARs, which are inherited just like autosomal chromosomes) on chromosomes X and Y, as defined by genomic positions in the current genome builds. The genomic annotation of the PAR region has undergone a series of changes throughout different PLINK versions. In earlier versions, PLINK stored both PAR1 and PAR2 variants separate from the X (23) and Y (24) chromosomes, namely, in a virtual chromosome 25, while newer PLINK versions use alphanumerical chromosome codes such as PAR1 and PAR2. Because not every tool is aware of these changes, they are often mixed up in chromosomes 23, 24, 25, or all of them. The Pre-QC phase consistently moves both PAR1 and PAR2 variants back into chromosome 25 according to the coordinates published by the Genome Reference Consortium for GRCh37 [22]; after the QC, chromosome 25 variants are moved back to chr23 for the generation of the separate imputation VCF input files because this is required by the TOPMed Imputation Server (see section on VCF output). If a data set presents heterozygous calls for males outside the PAR1/PAR2 regions and, hence, belongs to the non-PAR regions on chromosomes X or Y, heterozygous genotype calls for males are likely to be sequencing errors and will be set to “missing." The QC Module does not perform a check between self-reported and genetically determined biological sex based on chromosome X PLINK genotypes; we recommend using normalized signal intensities of SNPs on chromosome X as well as Y for this purpose.

#### Automatic chip type and variant annotation detection

Because many GWAS platforms (microarray or chip type) have specific properties and exhibit potential biases, it is crucial for the QC to know the exact chip type from which the data set was generated. This step helps in identifying the chip type by matching chromosome IDs, variant IDs, base-pair positions, and alleles of variants from PLINK’s bim files against a set of currently 140 original GWAS platform content information files (called strand files) provided by Illumina and Affymetrix [23]. The results of the chip detection step are expressed as match rates (in percentages) and can be used to verify not only the true chip type but also whether variant identifiers and/or the string information have already been manipulated by the user. Our current chip detection database contains 140 different chip types (including genome builds 37, 38, and partially 36), resulting in 415 chip versions that can be detected. The strand files are processed by a configurable number of threads (per default 2 threads only), where each thread processes a strand file as follows: first, every file entry (i.e., variant) of PLINK’s bim file is matched against the strand file using variant ID, positition, and alleles. After the final variant match, a composite match rate is calculated to assess how well a particular strand file (or chip type) matches the annotation in the bim file. After each string file is processed, the list of chip matches is sorted by a weighting formula, with *m* denoting the respective match rate:

\begin{eqnarray*}
m_{\text {sort}} = m_{\text {ID}}\cdot m_{\text {pos}} \cdot \mathrm{max}\left \{ m_{\text {orig}\_ {\text {stand}}} , m_{\text {plus}\_ {\text {stand}}}\right \}. \end{eqnarray*}

It is often the case that variants are converted to the plus strand before QC. To make the match sorting robust, both the allele match rate for the original strand definitions and the allele match rate for the plus strand will be calculated. The strand files are then sorted by *m*_sort_ and can be used later for an optional genome build liftover process (non-hg19 data and liftover to hg19). If the bim files match the plus strand better than the original strand information, a note is displayed in the PDF report. For performance reasons, the detection software has been written in the Rust programming language and is also available as a separate application (see Section Availability of Source Code and Requirements).

#### Automatic genome build liftover of genomic coordinates

Currently, the QC pipeline only supports data on genome build 19 (or GRCh37). If input data are in a different format, e.g., hg18 (GRCh36) or hg38 (GRCh38), the liftover step can be explicitly enabled to perform an automatic liftover to hg19. If the chip type is known beforehand or from the automatic chip type detection step (see above), a liftover request can be set in the data set configuration file. A genome build conversion is often accompanied by the loss of some variants when variants in the target build are split into ≥2 variants or merged into 1. This may cause unmappable or duplicate variants that will subsequently be removed from the data set. A warning will be displayed in the PDF report if a liftover is requested and more than a pre-defined number of variants are lost due to this step (defaults to 10 %).

#### Matching of variant identifiers with external databases

Different GWAS platforms often use different variant identifiers for the same variants. In this step, variant identifiers from PLINK’s bim files are matched against a variant database containing identifiers of the Haplotype Reference Consortium (HRC) 1.1 reference panel [24], the UK10k cohort [25], the 1000 Genomes Phase 3 Project [26], and the db150 variant database [27]. If a single unique match is found, the respective variant name is retrieved and the original name replaced. In case the variant alleles match the strand complement of the reported alleles in the database, the variant is scheduled for strand alignment in a later step. Some genomic positions are covered by >1 variant, i.e., deletions that span multiple positions and overlap with SNPs, so that it is not clear which variant is to be assigned. In these cases, the name is set to the chr:pos format, e.g., 1:554127. If no match could be found in the database, the original variant name is retained and prefixed with “unk_" to indicate that neither name, position, alleles, nor strand alignment could be verified, which is often the case if the content is custom chip content. Variants with different names but same position and same alleles are classified as duplicates and 1 of the duplicates is discarded afterwards.

### SNP QC I

During SNP QC I, the prepared data sets, which were pre-processed in parallel in the pre-QC phase, are merged into a single data set and filtered by missingness and HWE (in controls only) across all batches as well as single batches from a particular phenotype and the control group. DeFinetti diagrams [28] are created before and after SNP QC I to verify the quality of variants visually.

#### Merge step

If several input data sets have been defined, they are merged into a single PLINK data set. However, the samples will still be analysed taking into account the previously defined batches.

#### Missingness

Variants with a high genotyping error rate or which are only present in a subset of the data sets may now be discarded on the basis of variant missingness. Variant missingness is calculcated across all batches as well as single batches. The default missing call rate thresholds are 0.1 and 0.02, respectively. If desired, these can be reconfigured by the user.

#### Tests for Hardy-Weinberg equilibrium

HWE tests are performed for controls only in a case-control study (and for all individuals if a quantitative trait is to be considered). HWE tests are performed for all variants from the autosomes (chromosomes 1–22) and the PARs. For women, the complete chromosome X is also tested if available. Per default, variants deviating from HWE (with an FDR threshold of 10^−5^ in controls per default) across the entire batch collection with at most 1 batch being removed or falling below the FDR threshold in 2 single control batches are excluded. Batch-wise *P*-values are reported to find variants that fail HWE, among others, in a single control batch (but not in other control batches), in ≥2 control batches, and in the whole collection of controls. Because this version of HWE testing is a computationally intensive task, the data set is divided into small pieces of 1,000 variants each, which are then processed in parallel if the surrounding compute environment allows it (e.g., on an HPC cluster). Diagrams are also generated showing how many variants are removed in each specific case; if desired, the user can adjust the threshold parameters using these diagrams.

### Sample QC

After SNP QC I, several sample QC measures will be calculated to identify samples showing a high variant missingness rate or an increased or decreased heterozygosity rate, to identify duplicate and related samples and to examine potential population substructure or batch effects by means of principal component analysis (PCA) and Tracy-Widom statistics, with reference samples from HapMap and the 1000 Genomes Project.

#### Individual missingness and heterozygosity

In this step, a list of sample outliers is generated through a series of computations that determine the sample missingness of each data set and the sample-wise heterozygosity rates across all variants. For the former characteristic, the PLINK tool is run to generate a summary statistic of the genotyping rates per individual sample. Per default, samples that have a missing rate of ≥0.02 are removed. If desired, the user can adjust this threshold. The heterozygosity is determined with a second PLINK call, comparing the expected homozygosity rate with the observed homozygosity rate. Samples with a heterozygosity rate of ±5 × standard deviation are classified as outliers. A scatter plot of the proportion called missing (x-axis) against the proportion of SNPs called heterozygote (y-axis) for each individual in the study is generated.

#### Controlling for population stratification and other confounders

The most common method for identifying (and subsequently removing) individuals with large-scale differences in ancestry is PCA. To resolve within-Europe relationships (the default) and to test for population stratification, all samples are projected onto PC axes generated from 4 intercontinental HapMap populations as well as 26 reference populations from the 1000 Genomes Project using FlashPCA [29]. The distribution of cases and controls is displayed along the first 10 PCs stratified by phenotype. Individual GWAS samples (grey crosses) with values smaller than the median ±5 times the interquartile range (median ±5 × IQR; samples outside the red square in PCA plot) represent PCA outliers for the first 2 PCs and are automatically removed (the default; adjustable). All remaining samples (without PCA outliers) are projected onto PS axes generated from 26 reference populations from the 1000 Genomes Project again. Samples are also visualized without reference population samples using FlashPCA, with samples colored by batch code, and the distribution of cases and controls is displayed along the first 10 PCs stratified by phenotype. Tracy-Widom statistics [30] are further computed with EIGENSOFT/EIGENSTRAT (RRID:SCR_001357) [31] to evaluate the statistical significance of each PC identified by PCA and to decide which top axes of variation are significant and should be used as covariates in association analyses. For each PCA, a set of independent (minor allele frequency [MAF] >0.05) SNPs is calculated, excluding non-autosomes, variants in linkage disequilibrium (LD) (leaving no pairs with *r*^2^ > 0.2, within 50-kb windows) or within the extended major histocompatibility complex (xMHC; chr6:25-34Mb), and 11 high-LD regions as described by Price et al. [32]. PCA plots with and without A/T and G/C SNPs are created to reveal potential undetected strand problems.

Population codes from the 1000 Genomes Project are as follows: African; Ad Mixed American; East Asian; EUR, European; SAS, South Asian; CHB, Han Chinese in Bejing, China; JPT, Japanese in Tokyo, Japan; CHS, Southern Han Chinese; CDX, Chinese Dai in Xishuangbanna, China; KHV, Kinh in Ho Chi Minh City, Vietnam; CEU, Utah Residents (CEPH) with Northern and Western Ancestry; TSI, Toscani in Italy; FIN, Finnish in Finland; GBR, British in England and Scotland; IBS, Iberian Population in Spain; YRI, Yoruba in Ibadan, Nigeria; LWK, Luhya in Webuye, Kenya; GWD, Gambia; MSL, Mende in Sierra Leone; ESN, Esan in Nigeria; ASW, Americans of African Ancestry in SW USA; ACB, African Caribbeans in Barbados; MXL, Mexican Ancestry from Los Angeles, CA, USA; PUR, Puerto Ricans from Puerto Rico; CLM, Colombians from Medellin, Colombia; PEL, Peruvians from Lima, Peru; GIH, Gujarati Indian from Houston, TX, USA; PJL, Punjabi from Lahore, Pakistan; BEB, Bengali from Bangladesh; STU, Sri Lankan Tamil from the UK; ITU, Indian Telugu from the UK.

#### Duplicate/relatedness detection

For robust duplicate/relatedness testing (identity-by-state [IBS] and IBD estimation), we use the same set of independent variants, which was dynamically determined for PCA analysis (see above). Pairwise percentage IBD values were computed from a pruned subset of independent SNPs (see above) using PLINK. By definition, $P(\mathrm{IBD} = 0) + P(\mathrm{IBD} = 1) + P(\mathrm{IBD} = 2) = 1$and $\hat{\pi }:= P(\mathrm{IBD} = 2) + 0.5 \cdot P(\mathrm{IBD} = 1)$ (proportion IBD). The expectation is that IBD = 1 for duplicates or monozygotic twins, IBD = 0.5 for first-degree relatives, IBD = 0.25 for second-degree relatives, and IBD = 0.125 for third-degree relatives. Owing to genotyping error, LD, and population structure, there is often some variation around these theoretical values and it is typical to remove 1 individual from each pair with an IBD value of >0.1875, which is halfway between the expected IBD for third- and second-degree relatives. For these reasons an IBD value of >0.98 identifies duplicates (adapted from [15]). One individual (the one showing greater missingness) from each pair with $\hat{\pi }> 0.1875$ is flagged in output files or is removed.

For kinship detection, by definition, every possible pair of samples is analysed. The expected runtime is therefore quadratic with respect to the number of samples. With recent data sets growing past 500,000 samples, such as the UKB Data Release [2], this becomes a computationally intensive task, almost impossible to process on a single computer in an acceptable time frame. Our pipeline software automatically splits the workload and distributes the individual tasks to the available compute nodes in an HPC environment, if available, to process biobank-scale data. With the current, user-configurable scaling settings and assuming that all split jobs can be run in parallel, the kinship detection would only take as much time as a 2,000-sample data set would take, even for large sample databases.

### SNP QC II

After Sample QC, a few final SNP QC measures are calculated to identify variants where batches within a phenotype and the control group have different allele frequencies even after correction for potential population substructure and variants that show significantly different missingness between cases from the same phenotype and controls.

#### ANOVA Test

Significantly different allele frequencies of variants across the batches from a particular phenotype or the control group due to batch effects can be detected via ANOVA that adjusts for potential population structure from PCA analysis. This tests for difference in the mean allele frequencies (after correcting for population stratification) across different batches from a particular disease or the control group. First, allele dosages are regressed against top PCs from PCA and residuals were obtained. *P*-values are calculated from the ANOVA between the residuals of the genotypes and the batches. The null hypothesis is that all groups from a particular disease or from the group are simply random samples of the same population. Variants that have significantly different allele frequencies across the batches (with allowing a single batch to be removed) within phenotypic sets with an FDR threshold of 0.01 (the default) are removed. Robust detection of outlier variants is only possible with the availability of several different batches for the same diagnosis. Hence, this step is only performed if there is ≥1 defined diagnosis with ≥5 batches.

#### Differential missingness

Given a case/control phenotype, the task is to detect platform/batch differences between case and control genotype data that may lead to false-positive signals in association analysis. A Fisher exact test on case/control missing call counts at each variant will be performed. Any variant that comes up as highly significant under this test (*P* ≤ 10^−15^ per default) should be treated with great caution and will be excluded.

### Final analysis and report generation

In this stage, the final quality controlled output has been generated as no more variants or samples will be removed on the basis of quality metrics. To visualize the changes resulting from the removal of variant and sample outliers, a summary based on several PCA plots is generated.

#### Variant call format output

The quality controlled GWAS data set is also converted to VCF so that it can be used as input for genotype imputation and association testing tools. Data sets are converted to the VCF 4.2 format [33] and split to chromosome-wise files as expected by many imputation tools such as the TOPMed Imputation Server [20].

#### Automatic liftover to hg38 (optional)

The final VCF files are currently based on genome build hg19. Services such as the TOPMed imputation server [20] automatically convert VCF files to hg38, making it difficult to merge post-imputation data with pre-imputation data sets prepared in hg19. Optionally, the conversion to hg38 can be performed automatically by the pipeline using the UCSC liftOver Tools [34]. Owing to licensing issues, the UCSC liftover tools [35] are not directly included in our implementation but can be easily connected to our established UCSC liftover interface with a few steps. The same set of VCF files as for hg19 is generated in hg38 to facilitate the use of downstream imputation and association testing toolchains.

#### PDF report generation

Many other pipeline tools that provide GWAS QC include a number of program log files and other logs from which the number and IDs of removed variants and samples must be extracted for a final report. This can be a laborious and error-prone process. In our software, we introduce the automatic generation of a sophisticated PDF report that visualizes all changes and analysis of input data sets in plots and tables within a well-structured, single PDF document. A large collection of figures and tables before and after the QC are arranged side by side to allow easy verification of the results. Using the Nextflow engine, all process executions are further recorded in a single trace file. Our reporting software parses this trace file into an “execution tree," where all generated files, such as log files, program output, and graphics, can be accessed directly by their task name. A collection of custom parsers then uses the execution tree to create a template in the LaTeX typesetting language, which is finally compiled into a PDF file. A working LaTeX installation is not required because all necessary software is included in the accompanied Singularity image.

### Association testing module

**Figure 3:**
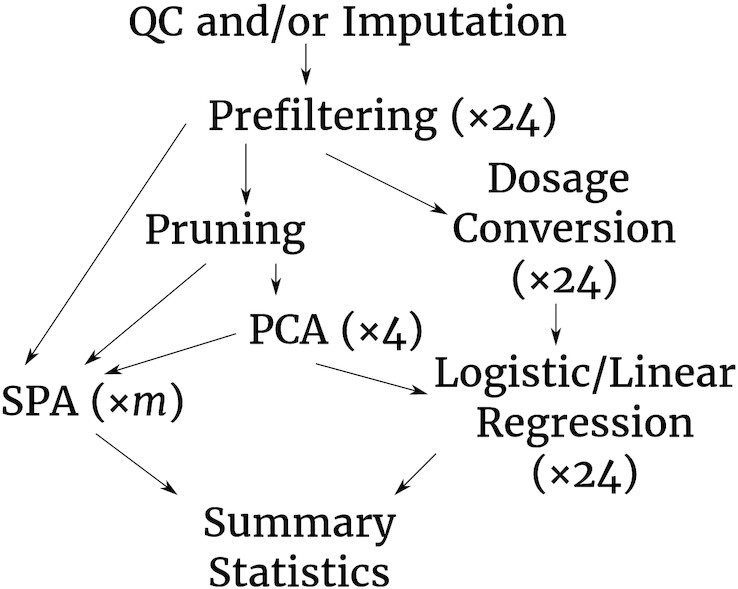
Workflow graph of the Association Testing Module of BIGwas, with default number of threads used in each process (for details see Methods). For example, association analysis is parallelized over a fixed number of chromosomes (×24). *m* denotes that the number of threads linearly scales with the number of variants. PCA: principal component analysis; SPA: saddle point approximation to calibrate unbalanced case-control ratios (see Methods).

Association analysis in GWAS/PheWAS is used to identify variants and regions of the genome associated with the phenotype of interest or to examine variants of special interest for their impact on a large collection of phenotypes. The most used software tools for single-variant association analysis are PLINK [6] and SAIGE [21], among others. Logistic/linear regression as implemented in PLINK is the de facto gold standard for association analysis, with logistic/linear mixed models as implemented in SAIGE now being the new standard for biobank-scale GWAS and PheWAS. The Association Testing Module allows association analysis for binary and quantitative traits with PLINK and SAIGE on both imputed and non-imputed GWAS data sets on different genome builds, both with and without covariates, including chromosome X analysis, combined in a single command. For quantitative traits, the user has to set the parameter “-trait" to “quantitative” instead of “binary” (the default). In this case, PLINK as well as SAIGE will then automatically perform an association analysis for a quantitative trait. All processes, including conversion steps, are largely processed in parallel, so that each GWAS association analysis for each phenotype can be accomplished by a single run (see Fig. 3).

### Data preparation

No further genotype data preparation step is required if the input data set, a set of chromosome-wise VCF files (optionally including chromsome X), is obtained either from the QC Module or from genotype imputation programs such as the TOPMed Imputation Server and contains dosage and/or genotype information. VCF files must have a DS tag for dosage data or a GT tag for genotyped data. If both are present, DS is chosen.

### Sample selection, covariate preparation, and null model building for SAIGE

To select a desired subset of samples for association analysis, an optional PLINK FAM file to update sex information and case-control status can be specified, and an optional covariate file if covariate adjustment is desired. In this case only the joint set of samples from the sample list of the VCF and the optional FAM file (and the optional covariate file) is used for model building (in SAIGE) and association analysis; no further modification of the VCF input files is necessary. For model building in SAIGE using saddle point approximation (SPA) [21] to calibrate unbalanced case-control ratios, the null logistic/linear mixed model is fitted on the basis of a set of independent variants (see section Controlling for population stratification and other confounders, Methods), with all independent variants also having an imputation quality score of *r*^2^ ≥ 0.8 (threshold configurable). With the same set of independent variants PCA analysis is performed using FlashPCA2 [29] to calculate top PCs (default n = 10) to adjust for potential population stratification.

### PLINK association testing

PLINK’s logistic/linear regression association module requires a custom text-based file with genotype dosage information (genotype probability format), which is generated on the fly from imputed or non-imputed VCF input files. Genotype dosage–based association analysis is conducted with the top PCs (default n = 10) from PCA and user-defined optional covariates from the covariate file. For chromosome X association analysis, haplotypic allele calls outside the PAR regions in males are converted to homozygous calls by doubling the haplotypic allele (assuming inactivation of large parts of 1 of the 2 female X chromosomes [36]), and sex is used as a covariate for association testing of the non-PAR regions on chromosome X.

### SAIGE association testing

One of the main features of SAIGE is its ability to perform single-marker association testing for extremely unbalanced case-control ratios and rare variants [21]. While SAIGE’s computational load is comparable to logistic or linear regression as performed by PLINK, SAIGE can directly read compressed VCF files typically generated from genotype imputation programs, including imputed dosage data and genotype-only calls, and further supports the memory-efficient BGEN format as used in the UK Biobank imputed data releases. SAIGE allows much finer control on parallelization by allowing the association analysis on specified genomic parts of a VCF/BGEN without the need to cut the file into smaller chunks. This technique saves not only runtime that would otherwise be consumed by splitting up VCF files but also large amounts of temporary file storage and I/O load. Because no extra computational load is required for splitting of chromosomal files, the scalability of how many jobs can be executed in parallel is only limited by the available computational resources, and the overhead is very low with respect to the chunk size of the genomic parts. Using only a subsample of all samples from VCF input files is not yet supported by SAIGE. For this reason, we have written a routine that automatically extracts a subsample from the VCF files based on a user-defined PLINK FAM file before starting the analysis with SAIGE. Association analysis with SAIGE (mixed-effect model + covariates) is conducted using the top PCs (default n = 10) from PCA and user-defined optional covariates from the covariate file. The same chromsome X analysis method used by PLINK is performed in SAIGE.

### Compatibility with UK Biobank data

The UKB’s latest GWAS data release contains, in addition to unimputed genotype data in PLINK format, pre-imputed data in the BGEN format instead of the much more common VCF format. However, most computational procedures of the Association Testing Module, especially the conversion routines that convert VCF files to PLINK’s genotype dosage format, heavily rely on the VCF format specification. BGEN, while much more efficient than VCF, is a binary format that is not designed to be processed by scripts and, as such, cannot directly be processed into PLINK’s genotype dosage format. Because the more common VCF format is a generic format used by biobanks and genotype imputation programs (TOPMed and Sanger Imputation server  [20], among others) worldwide, we chose to drop BGEN support and therefore, in addition to SAIGE, can still keep PLINK as a de facto gold standard tool for regression-based association analysis in our association testing workflow.

However, we included a set of scripts to perform SAIGE association testing on UKB BGEN files. This “pipeline” is a feature-reduced version that only performs the absolutely necessary steps to run SAIGE on the input data but uses the same fine-grained parallelization techniques as used in the SAIGE/PLINK VCF-based pipeline. External covariates, such as PCs, sex, and age, can be extracted from UKB sample information databases and supplied to the association testing process.

### Implementation details

We use the free and open-source Nextflow [13] pipeline software for easy parallelization of processes, HPC-based development, and pipeline control. Singularity, a container format and software focused on scientific computation and reproducibility, is used to provide a single-command experience without the need to install an entire software execution environment of different software packages.

### Nextflow

While both the QC Control Module and the Association Testing Module are on the same level of runtime performance as comparable GWAS pipelines that analyse a smaller number of samples and variants (cf. Tables 2 and 3), the real challenge of existing GWAS pipelines becomes apparent with larger data sets. For example, many QC and association testing tools use the duplicate/relatedness detection feature (see Methods) to remove related individuals or duplicate samples from the data set. Because every sample is tested against every other sample, the total runtime grows quadratically in relation to the number of samples; i.e., the time complexity is $\mathcal {O}(n^2)$.

Nextflow, a tool specifically designed to run bioinformatics applications, therefore provides a suitable environment to facilitate the parallelism of processes in all workflows. It extends the Unix pipes model with a fluent data descrition language (DSL), allowing the user to handle complex stream interactions easily. In our case, we use a dynamic job creation approach to automatically determine the number of parallel jobs required to perform an operation within a reasonable time frame. For example, in the duplicate/relatedness detection step, we use both PLINK’s “-parallel” mode and Nextflow’s job splitting feature to achieve fast processing of large input files. The same mechanism is also used for other computationally intensive operations, such as our “leave-one-batch-out Hardy-Weinberg test” or the SAIGE association testing procedure. Nextflow takes care of the creation, workspace separation, and merging of all jobs, avoids user-defined error-prone synchronization of all jobs, and oversees the merging of results from complex analysis pipelines. All the intermediate results produced during the pipeline execution are automatically tracked.

Another major advantage of Nextflow is the already integrated handling and agnostic use of HPC cluster resources (i.e., it is able to work under different environments). With Nextflow, it is possible to write parallel and concurrent pipeline workflows without having knowledge (and thus knowing all dependencies) of a specific cluster environment, such as SGE, SLURM, or PBS/TORQUE. This makes it possible to provide pipelines for a wide range of HPC and non-HPC users. The deep integration of different storage solutions like ECS object storage or S3 buckets and container tools such as Docker and Singularity make Nextflow a first choice for a portable, widely available platform, with the only requirement of a working Java installation.

### Singularity

Our general Nextflow script code base is hosted on GitHub. However, the included pipeline scripts make heavy use of external tools, such as the well-known PLINK sofware  [6] for GWAS data. Because each workflow is usually created with specific versions of tools in mind, it is often the case that developers need to install multiple versions of the same software and must take special care in managing them. Conda (RRID:SCR_018317)  [37] and Lmod [38] assist in setting up the respective software environments, but the required tools must still be installed manually by the user.

In our case we decided to prepare the execution environment, including an entire operating system, in a publicly hosted container image, including all specific program versions. This not only eliminates the burden and error-proneness of manually creating a new environment but also ensures that each user uses exactly the same program versions and therefore can reproduce any result with the same input, without any problems with deployment on different operating systems, user permissions, or configuration management.

Another advantage of using an entire file system is the possibility not only to add programs as desired but also to include any file. We use these capabilities to provide ready-to-use Hapmap2 [39] and 1000 Genomes Project [26] data sets that serve as reference samples, e.g., for PCA, as well as a comprehensive but slim database of NCBI Reference SNP ID numbers [40].

## Results

### Benchmark settings

In Table 1 we compare the features of the QC Module of BIGwas (for details see Methods) with the features of the QC module from the H3Agwas Pipeline Version 3, a portable and reproducible GWAS QC and association test software workflow developed by the pan-African community project H3ABioNet (see Introduction). The H3A QC module was developed to perform a robust and efficient QC workflow for >30,000 DNA samples genotyped using a custom-designed African genotyping array as part of H3ABioNet [12]. Because many similar features were implemented in both software pipelines (although there is no explicit support for multiple GWAS data sets [i.e., batches] in H3A QC), the runtime performance can be compared very well in a benchmark. We have not made a feature comparison for the association pipelines of BIGwas (for details see Methods) and H3A because the authors of the H3A Association Pipeline have made it clear that the purpose of the H3A Association Pipeline is to perform a very basic logistic/linear regression association analysis with PLINK [41]. The use of linear mixed models for association testing is further supported but only for genotypes in PLINK format (and not imputed data in VCF format as delivered by genotype imputation servers) and without support for pre- or post-processing of input and output files. For this reason, we only compared the runtime of logistic/linear regression and mixed model testing with BIGwas (PLINK and SAIGE) versus logistic/linear regression with H3A (PLINK).

**Table 1:** Feature comparison between the QC Module of BIGwas and the H3A QC module of the H3Agwas Pipeline Version 3  [12]

Feature	BIGwas QC	H3A QC
Duplicate Removal	✓	✓
RsID dbSNP Assignment	✓	✗
HWE Test	✓	✓
HWE Test (batch-wise leave-one-out)	✓	✗
IBD/Relatedness Check	✓	✓
Missingness Check	✓	✓
Heterozygosity Check	✓	✓
PCA Outlier Detection	✓	✗^1^
Low-MAF Removal	✗	✓
PAR Check	✓	✗
VCF Output	✓	✓
Genome Build Liftover	Optional	✗
Dynamic Parallelization of Jobs	✓	✗^2^
PDF Report	✓	✓

1 Principal component analysis (PCA) is calculated but ancestry outliers are not determined.

2 Parallelization effort is manually configurable but does not automatically scale.

For details see Methods. The QC Module of BIGwas performs, among other things, an automatic upscaling of job parallelization with respect to the number of samples and variants and allows an unlimited number of input GWAS data sets (i.e., batches) to be processed with a single command. HWE: Hardy-Weinberg equilibrium; MAF: minor allele frequency; PAR: pseudo-autosomal region; PCA: principal component analysis.

We conducted benchmark tests on our HPC system with 68 computational nodes and 1,336 CPU cores in total, which enables highly parallel computing. However, because in a multi-user HPC system not all resources are usually available to a single user, and in order to demonstrate that BIGwas uses resources economically, for benchmarking we allowed the submission of a maximum of 150 jobs in parallel (corresponds to 7 compute nodes; configurable by the user) for testing both the QC Module and the Association Testing Module of BIGwas. For benchmarking H3A, we made no restrictions on HPC resources (thus allowing 1,336 jobs in parallel) to test the maximum performance of H3A. For each benchmark data set consisting of a different number of GWAS samples and genetic variants (Tables 2 and 3), we used Nextflow’s built-in reporting function to determine the elapsed wall-clock time. Each runtime analysis was performed 3 times on an empty HPC cluster to account for potential fluctuations in HPC cluster performance.

**Table 2:** Runtime metrics of the QC module of BIGwas compared to the QC module of the H3Agwas Pipeline Version 3  [12] for several different-sized GWAS data sets

Sample No.	Variant No.	BIGwas Runtime	H3A Runtime
200	500	6 min	4 min
500	1,000	6 min	7 min
1,000	5,000	6 min	9 min
5,000	50,000	8 min	18 min
5,000	250,000	15 min	35 min
10,000	250,000	15 min	57 min
20,554	700,078	2 h 15 min	8 h 57 min
488,292	231,151	3 d 1 h	*
488,292	803,113	4 d 22 h	*
976,584	803,113	6 d 12 h	*

A GWAS input data set with 976,584 samples and 803,113 genetic variants (i.e., twice the size of the current UK Biobank GWAS data set) can be quality-controlled in <7 days with 1 command of the BIGwas software, whereby only 150 jobs (configurable; equivalent to ∼7 compute nodes on our HPC cluster system) are used in parallel. *Runtime of H3A has exceeded the maximum time allocation of 10 days for the HPC system despite the possibility of using a maximum of 1,336 jobs in parallel.

**Table 3:** Runtime metrics of the Association Testing Module of BIGwas (regression analysis with PLINK and mixed model analysis with SAIGE) compared to association testing module of the H3Agwas Pipeline Version 3 (regression analysis with PLINK) [12] for several different-sized GWAS data sets

Sample No.	Variant No.	BIGwas Runtime	H3A Runtime
10,000	250,000	21 min	8 min
10,000	700,078	54 min	16 min
20,554	700,078	1 h 33 min	1 h 23 min
5,480	81,708,012	14 h 26 min	*
487,409	92,775,302	4 d 18 h	*
974,818	92,775,302	9 d 14 h	*

An imputed genome-wide input data set with almost 1 million samples and >92 million genetic variants (i.e., twice the size of the imputed UKB GWAS data set) can be tested for association within 10 days with 1 command of the BIGwas software, whereby only 150 jobs (configurable; equivalent to ∼7 compute nodes on our HPC cluster system) are used in parallel. *H3A uses PLINK genotype files as input (but not imputed allele dosages).

### Benchmark results

Table 2 shows runtime benchmarks of the QC Module of BIGwas (for details see Methods) compared to the QC module of the H3Agwas Pipeline Version 3. For comparison with H3A runtimes, we set configurable parameters, such as missingness thresholds, to similar values where possible and used the recommended commands for the respective benchmark data sets. Data sets in the upper part were subsampled from in-house GWAS data sets gentoyped using Illumina’s Global Screening (GSA) Array v1.0. In the lower part, unimputed Axiom Array data of 488,292 individuals of European ancestry from UKB were used (see Acknowledgements). The data set of 976,584 samples and 803,113 SNPs represents an artificially created GWAS data set (from the original COVID-19 UK Biobank unimputed GWAS data release, September 2020, with 1,485 COVID-19 cases and 486,807 controls), which was created by duplicating the number of samples from the UK Biobank data set.

The theoretical algorithmic runtime complexity of the QC is quadratic with respect to the number of samples and linear with the number of variants $\mathcal {O}(n^2+m)$ because in IBD analysis each sample is checked against every other sample both in the H3A as well as BIGwas. The IBD calculation becomes the most time-consuming part from 5,000 samples upwards in H3A QC and leads to a preliminary termination of H3A QC for the UKB data set (Table  2). For 5,000 GWAS samples upwards, our implementation begins with a dynamic parallelization process (see Methods), i.e., scheduling (at maximum 150) jobs (configurable) to distribute the workload across multiple compute nodes in order to reduce the runtime (not shown) with respect to the number of samples. The number of additional jobs scales with the number of samples to keep the runtime for IBD analysis low (see processes marked with ×*n* in Fig. 2); thus the runtime of BIGwas increases much slower than that of H3A. Algorithmic complexity in other time-consuming applications such as Hardy-Weinberg and ANOVA testing is generally linear in the number of variants, but we found it useful to also scale the number of parallel jobs or threads automatically with the number of samples (also marked with ×*n* in Fig. 2). Our resource-efficient intra- and inter-node software implementation (see Methods) shows that BIGwas did not exceed the maximum number of 150 parallel jobs allowed by us in our benchmark and that BIGwas is able to analyse arbitrarily large GWAS input data sets with 1 million GWAS samples. Despite the possibility of using a maximum of 1,336 parallel jobs, H3A exceeded a runtime of 10 days for the UKB data set with 488,292 samples and a reduced set of 231,151 (LD pruned) variants (Table  2). Results of BIGwas are summarized in a PDF report (see Methods), and output files (on either genome build hg19 and/or hg38; configurable) can be used directly for association testing and/or as an input for genotype imputation services.

Table 3 shows runtime benchmark results of the Association Testing Module of BIGwas (for details see Methods) compared to the association module of H3A. Data sets in the upper part were subsampled from in-house GWAS data sets gentoyped using Illumina’s Global Screening (GSA) Array v1.0. In the lower part, pre-imputed GSA data of 5,480 individuals as well as pre-imputed Axiom Array data of 487,409 individuals of European ancestry from UKB were used (see Acknowledgements). The data set of 974,818 samples and 92,775,302 SNPs represents an artificially created imputed GWAS data set (from the orginal COVID-19 UK Biobank imputed GWAS data release, September 2020, with 636 cases and 486,773 controls), which was created by duplicating the samples of the UKB data set. Association analysis included top 10 PCs from PCA, sex, age, sex*age, and age*age as covariates.

The theoretical runtime of BIGwas increases linearly with the number of variants $\mathcal {O}(m)$. The runtimes shown in Table 3 were achieved with a maximum number of parallel jobs of 150 for BIGwas, and with no restrictions on the number of parallel jobs for H3A (at maximum 1,336 jobs in parallel). By default, our pipeline module schedules 1 job for 5,000 genetic variants (marked with ×*m* in Fig. 3) for the limited number of maximum 150 parallel jobs (configurable). Our resource-efficient intra- and inter-node software implementation (see Methods) enables BIGwas to analyse arbitrarily large GWAS input data sets with millions of GWAS samples and millions of imputed genetic variants. Although SAIGE and PLINK association testing in BIGwas is performed in parallel in 1 run of the Association Testing Module, the runtime of BIGwas is only slightly longer than that of H3A, which performs association testing with PLINK only, without pre- and post-processing of input and output files. Some new features have been implemented in the Association Testing Module of BIGwas, which are not covered by the original implementations of SAIGE and PLINK (see Methods): before association tests are performed, an on-the-fly sample filtering is conducted to exclude certain samples (e.g., based on a sample exclusion list; see Methods) or variants (e.g., based on the imputation score threshold *r*^2^; see Methods) from PLINK or VCF input data sets, without the need to manually remove them first, which is usually a labor-intensive step for the user. In addition, an automated PCA (configurable) is integrated into SAIGE mixed-model association analysis, so that model fitting analysis and association testing (along with PCs from PCA and/or user-provided covariates) are performed in SAIGE in a single step; association testing for chromosome X is also automated for PLINK and SAIGE (see Methods). Association summary statistics output files as well as best-guess genotype calls in PLINK format are produced for different genome builds (hg19 and/or hg38; configurable).

## Discussion

We have introduced BIGwas, a portable (i.e., Singularity container-based), fully automated GWAS QC and association pipeline in Nextflow that can be executed with a single command without manual installation by the user. BIGwas differs from existing GWAS data-processing packages in R in that it allows standardized, reproducible, and high-throughput processing of GWAS data sets with a single command, whereas existing GWAS R packages allow for more flexible processing, e.g., with respect to the order of processing steps. The current UKB GWAS release with 487,409 GWAS samples can be analysed with BIGwas within 8 days (4 days for QC and 4 days for association testing) on a small HPC with 7 compute nodes. For a GWAS data set with ≤1 million individuals BIGwas needs on average 16 days on an HPC with 7 compute nodes. Using our dynamic parallelization approach, shorter runtimes can be achieved with a larger HPC. For QC the theoretical runtime increases quadratically with the number of samples and linearly with the number of variants $\mathcal {O}(n^2+m)$. For association testing the runtime increases linearly with the number of variants $\mathcal {O}(m)$. As sample sizes in large GWAS/PheWAS biobank studies, e.g., for COVID-19 research [4, have grown to millions of GWAS samples and GWAS data sets of this size are likely to be used more frequently in the future, the efficiency and ease of execution of BIGwas with a single command will become an important criterion for conducting GWAS/PheWAS studies.

To enable a reproducible analysis of 1 million GWAS samples on any common compute platform, we have combined various code optimizations of our existing GWAS software with Nextflow workflow and Singularity container technology. Our Nextflow pipelines are inherently parallel and can be transparently scaled up or down, and they provide an abstraction layer between the logic of the pipeline and the execution layer (i.e., they can be executed on multiple platforms without changing the source code). With the Singularity container technology, we have packed all our Nextflow workflows, software, and libraries, as well as publicly available reference data, into a single Singularity container that runs on any standard computer platform without manual download or installation by the user.

BIGwas implements a complete GWAS analysis protocol that was developed for large-scale GWAS studies of the Inflammatory Bowel Disease Genetics Consortium and the Severe Covid-19 GWAS Group [16–18]. BIGwas is not yet suitable for GWAS analysis of family data or GWAS analysis across populations of worldwide ethnicities. To enable these analyses, extensions to our QC and the association testing pipelines or the use of alternative statistical methods may be required. In the future, we aim to test the usability of further genome-wide analysis approaches, such as gene-gene interaction tests, for use with Nextflow and Singularity technology.

## Availability of Source Code and Requirements

- Project name: IKMB GWAS QC and Association Testing Pipelines
- Project home page: http://github.com/ikmb/gwas-qc and http://github.com/ikmb/gwas-assoc
- Operating system: Linux
- Programming language: Nextflow, Bash, Python, Perl, R, Rust
- Other requirements: Java 8 or higher, Singularity 3.4 or higher
- Bio.tools identifier: https://bio.tools/bigwas_qc_pipeline and https://bio.tools/bigwas_assoc_pipeline
- RRID:SCR_019240 and RRID:SCR_019241

This project makes use of several open source tools, namely, PLINK 1.9 [42], PLINK 2 [6], bcftools 1.10 from the HTSlib project [43], FlashPCA 2.0 [29], EIGENSTRAT 4.2 [31], and SAIGE 0.42.1 [21]. For the availability of source code, we refer to the respective publications. Several more native binaries were created and included for performance reasons. Their source codes are available as follows:

- chipmatch, a tool to match PLINK’s BIM files against the Will Rayner strand database [23]: https://github.com/ikmb/chipmatch
- genericplotter, a high-performance heat map plotting tool for almost arbitrary, very large data sets: https://github.com/ikmb/genericplotter

UK Biobank (UKB) COVID-19 GWAS data release (September 2020): https://biobank.ndph.ox.ac.uk/ukb/exinfo.cgi?src=COVID19

## Data Availability

Snapshots of the code and example data sets are available in the GigaDB repository [44].

The example data set is a subset of the 1000 Genomes Project [26], using 2,504 samples with randomized sex and phenotype, and 50,000 randomly selected variants.

## Abbreviations

ANOVA: analysis of variance; FDR: false discovery rate; GWAS: genome-wide association study; HPC: high-performance compute; HWE: Hardy-Weinberg equilibrium; IBD: identity-by-descent; IBS: identity-by-state; LD: linkage disequilibrium; MAF: minor allele frequency; NCBI: National Center for Biotechnology Information; PAR: pseudo-autosomal region; PheWAS: phenome-wide association studies; QC: quality control; SNP: single-nucleotide polymorphism; SPA: saddle point approximation; UCSC: University of California Santa Cruz; UKB: UK Biobank; VCF: variant call format.

## Ethics Approval and Consent to Participate

All participants provided written informed consent, and the study was approved by the ethics boards of the participating institutions (see Acknowledgements) in agreement with the Declaration of Helsinki principles.

## Competing Interests

The authors declare that they have no competing interests.

## Funding

This work was supported by the German Federal Ministry of Education and Research (BMBF) within the framework of the Computational Life Sciences funding concept (CompLS grant 031L0165) and iTREAT (grant 01ZX1902A). The study received infrastructure support from the DFG Cluster of Excellence 2167 “Precision Medicine in Chronic Inflammation (PMI)” (DFG Grant: “EXC2167’’), the DFG research unit “miTarget” (Projectnummer 426660215), and the PopGen Biobank (P2N 2016-101).

## Authors' Contributions

J.C.K.: Conceptualization, Methodology, Software, Benchmarks, Writing. L.W.: Software, Benchmarks, Writing, Visualization. D.E.: Conceptualization, Methodology, Software, Formal analysis, Data curation, Supervision, Writing.

## Supplementary Material

giab047_GIGA-D-20-00360_Original_Submission

giab047_GIGA-D-20-00360_Revision_1

giab047_GIGA-D-20-00360_Revision_2

giab047_GIGA-D-20-00360_Revision_3

giab047_Response_to_Reviewer_Comments_Original_Submission

giab047_Response_to_Reviewer_Comments_Revision_1

giab047_Response_to_Reviewer_Comments_Revision_2

giab047_Reviewer_1_Report_Original_Submission Shing Wan Choi -- 12/23/2020 Reviewed

giab047_Reviewer_1_Report_Revision_1 Shing Wan Choi -- 4/20/2021 Reviewed

giab047_Reviewer_2_Report_Original_Submission Cristian Pattaro -- 1/2/2021 Reviewed
