# Supplementary material for: BIGwas: Single-command quality control and association testing for multi-cohort and biobank-scale GWAS/PheWAS data
Source: Gigascience. 2021 Jun 29;10(6):giab047. doi: 10.1093/gigascience/giab047 (PMC8239664; doi:10.1093/gigascience/giab047)
Supplement: giab047_GIGA-D-20-00360_Revision_2 [file giab047_giga-d-20-00360_revision_2.pdf]

GigaScience

BIGwas - Single-command Quality Control and Association Testing for multi-cohort and biobank-scale GWAS/PheWAS data

--Manuscript Draft--

**Manuscript Number:**	GIGA-D-20-00360R2
**Full Title:**	BIGwas - Single-command Quality Control and Association Testing for multi-cohort and biobank-scale GWAS/PheWAS data
**Article Type:**	Technical Note
**Funding Information:**	Bundesministerium für Bildung und Forschung (031L0165) — Dr. Jan Christian Kässens; Dr. Lars Wienbrandt; Dr. rer. nat. David Ellinghaus<br>Bundesministerium für Bildung und Forschung (01ZX1902A) — Dr. rer. nat. David Ellinghaus<br>Deutsche Forschungsgemeinschaft (DE) (426660215) — Dr. rer. nat. David Ellinghaus<br>Deutsche Forschungsgemeinschaft (DE) (EXC2167) — Dr. rer. nat. David Ellinghaus
**Abstract:**	Background<br>Genome-wide association studies (GWAS) and phenome-wide association studies (PheWAS) involving one million GWAS samples from dozens of population-based biobanks present a considerable computational challenge and are carried out by large scientific groups under great expenditure of time and personnel. Automating these processes requires highly efficient and scalable methods and software, but so far there is no workflow solution to easily process one million GWAS samples.<br>Results<br>Here we present BIGwas, a portable, fully automated QC and association testing pipeline for large-scale binary and quantitative trait GWAS data provided by biobank resources. By using Nextflow workflow and Singularity software container technology, BIGwas performs resource-efficient and reproducible analyses on a local computer or any high performance compute system (HPC) with just one command, with no need to manually install a software execution environment or various software packages. For a single-command GWAS analysis with 974,818 individuals and 92 million genetic markers, BIGwas takes around 16 days on a small HPC system with only seven compute nodes to perform a complete GWAS QC and association analysis protocol. Our dynamic parallelization approach enables shorter runtimes for large HPCs.<br>Conclusions<br>Researchers without extensive bioinformatics knowledge and with few computer resources can use BIGwas to perform multi-cohort GWAS with one million GWAS samples and, if desired, use it to build their own (genome-wide) PheWAS resource. BIGwas is freely available for download from http://github.com/ikmb/gwas-qc and http://github.com/ikmb/gwas-assoc.
**Corresponding Author:**	David Ellinghaus, Ph.D.<br>University of Kiel: Christian-Albrechts-Universitat zu Kiel<br>Kiel, GERMANY
**Corresponding Author Secondary Information:**	
**Corresponding Author's Institution:**	University of Kiel: Christian-Albrechts-Universitat zu Kiel
**Corresponding Author's Secondary Institution:**	
**First Author:**	Jan Christian Kässens, Dr.-Ing.
**First Author Secondary Information:**	
**Order of Authors:**	Jan Christian Kässens, Dr.-Ing.
	Lars Wienbrandt, Dr.-Ing.

Powered by Editorial Manager® and ProduXion Manager® from Aries Systems Corporation

	David Ellinghaus, Ph.D.
**Order of Authors Secondary Information:**	
**Response to Reviewers:**	Point-by-point reply for Submission ID GIGA-D-20-00360, “BIGwas- Single-command Quality Control and Association Testing for multi-cohort and biobank-scale GWAS/PheWAS data”<br><br>Reviewer Comments:<br><br>Reviewer #1:<br><br>In this update, the authors have addressed most of my concerns. In addition, the update to the README on their github sufficiently extensive, and I can now run the example data set after some minor changes. The paper should now ready to be published.<br><br>We thank the reviewer for the supportive feedback and the suggestions for modifications.<br><br>Some minor comment regarding the paper and the script:<br>1.Under "Assignment of variants to Pseudo-autosomal Regions", correct styling of "Plink" should be "PLINK"<br><br>We now write "PLINK" instead of "Plink" in all places in the manuscript.<br><br>2.The `qc-assoc-assets.sif` container is unsigned. As a result, singularity pull will fail and re-quire user to pull the singularity image themselves. Users will also need to set `singularity.cacheDir` variable in the ` ~/.nextflow/assets/ikmb/gwas-qc/nextflow.config` file. Not sure why setting this var-iable in the local nextflow.config doesn't work.<br><br>We now have created a signed version of the sif container image: https://cloud.sylabs.io/library/_container/5f43b624b1793638c11379be<br><br>In principle, Singularity also loads unsigned container files (default setting in Singularity). Generally it is not necessary to set the variable "singularity.cacheDir" in Nextflow, see https://www.nextflow.io/docs/latest/config.html#scope-singularity. However, we recommend to set the singularity cache directory in the configuration file $HOME/.nextflow/config, see also paragraph "Shared Singularity Cache" in our readme file at https://github.com/ikmb/gwas-qc/, to avoid having to download the image files over and over again. Please note that the cache directory must be accessible from all compute nodes when running BIGwas on a high-performance compute (HPC) cluster, see also paragraph "Shared Singularity Cache" in our readme file.<br><br>3.label 'phase' for multiple processes were not defined, causing pipeline to fail when using with HPC scheduler. It seems like `phase` should use at least 8 CPUs (required by flash-pca2_pruned_1kG process), but the default is 1.<br><br>We thank the reviewer for this valuable comment. The process you mentioned (flash-pca2_pruned_1kG) has indeed defined its own label to account for the CPU usage of flashpca2. However, the 'phase' label is reserved for the start of the phases (Rs,

Powered by Editorial Manager® and ProduXion Manager® from Aries Systems Corporation

	SNPQCI, SampleQC, etc.) and probably does not help with your pipeline crash. After reviewing the relevant source code, we have concluded that there may be either a configuration issue on your end or a problem with the pipeline control mechanism we are using. We would be happy to help you here and would appreciate it if you could post this issue in our public issue tracker at https://github.com/ikmb/gwas-qc/issues. It would also be very helpful if you could attach there 1) the ".nextflow.log" file generated by your crashed run, 2) the exact error message, and 3) the ".command.log" file located in the "working directory" that Nextflow shows in the error report, as well as 4) some details about your installation, for example, which job scheduler you are using, and how you have set up your personal Nextflow configuration.<br><br><br>Some suggestion that should:<br>-Considering that the HWE took up the most computation time, then a possibly quicker alter-native might be the following:<br>1.Run `--hardy` once in each cohort independently (or maybe --frqx for quantitative traits).<br>2.Extract the GENO column from the .hwe file to obtain the A1 hom, het and A2 hom count for the control samples<br>3.For each SNP, you should now be able to calculate the number of A1 hom, het and A2 hom count of control samples with all possible cohort combinations.<br>4.Use the standalone hwe p-value calculation software here: https://github.com/chrchang/stats to calculate the p-value for each SNP and cohort combina-tion<br>-The benefit of this approach is that the calculation of the A1, A2 and het count for each co-hort combination is now reduced to a single summation and the p-value should be able to be calcu-lated rather quickly. Hopefully this can help to speed up the current software<br><br><br>We thank the reviewer for this suggestion, which we will be happy to examine in more detail in the future. Such a major change to the code requires again extensive testing of the HWE subroutine for binary and quantitative traits, which is currently running without errors. Furthermore, we are currently using our implementation in R to additionally perform the HWE calculation and on-the-fly filtering for PLINK files with respect to the specified "ethnicity" from the annotation file, which has proven to be very effective to implement in R without having to further preprocess the PLINK files. Replacing the subroutine with a different source code also requires adapting the interface for generating the DeFinetti and HWE output figures. For this reason, we would like to leave the current implementation as it is.
**Additional Information:**	
**Question**	**Response**
Are you submitting this manuscript to a special series or article collection?	No
**Experimental design and statistics**<br><br>Full details of the experimental design and statistical methods used should be given in the Methods section, as detailed in our Minimum Standards Reporting Checklist. Information essential to interpreting the data presented should be made available in the figure legends.	Yes

Powered by Editorial Manager® and ProduXion Manager® from Aries Systems Corporation

Have you included all the information requested in your manuscript?	
**Resources**<br><br>A description of all resources used, including antibodies, cell lines, animals and software tools, with enough information to allow them to be uniquely identified, should be included in the Methods section. Authors are strongly encouraged to cite Research Resource Identifiers (RRIDs) for antibodies, model organisms and tools, where possible.<br><br>Have you included the information requested as detailed in our Minimum Standards Reporting Checklist?	Yes
**Availability of data and materials**<br><br>All datasets and code on which the conclusions of the paper rely must be either included in your submission or deposited in publicly available repositories (where available and ethically appropriate), referencing such data using a unique identifier in the references and in the "Availability of Data and Materials" section of your manuscript.<br><br>Have you have met the above requirement as detailed in our Minimum Standards Reporting Checklist?	Yes

Manuscript

Click here to access/download;Manuscript;BigWAS_manuscript_revisions2.

GigaScience, 2017, 1–12

doi: xx.xxxx/xxxx
Manuscript in Preparation
Technical Note

TECHNICAL NOTE

BIGwas- Single-command Quality Control and Association Testing for multi-cohort and biobank-scale GWAS/PheWAS data

Jan Christian Kässens[1,2*], Lars Wienbrandt[1] and David Ellinghaus[1,3*]

[1]Institute of Clinical Molecular Biology, Christian-Albrechts-University of Kiel, Kiel, Germany and [2]Haematology Lab Kiel, Klinik für Innere Medizin II, University Hospital Schleswig-Holstein, Germany and [3]Novo Nordisk Foundation Center for Protein Research, Disease Systems Biology, Faculty of Health and Medical Sciences, University of Copenhagen, Copenhagen, Denmark

[*]Corresponding authors, janchristian.kaessens@uksh.de, d.ellinghaus@ikmb.uni-kiel.de

Abstract

Background Genome-wide association studies (GWAS) and phenome-wide association studies (PheWAS) involving one million GWAS samples from dozens of population-based biobanks present a considerable computational challenge and are carried out by large scientific groups under great expenditure of time and personnel. Automating these processes requires highly efficient and scalable methods and software, but so far there is no workflow solution to easily process one million GWAS samples.
Results Here we present BIGwas, a portable, fully automated QC and association testing pipeline for large-scale binary and quantitative trait GWAS data provided by biobank resources. By using Nextflow workflow and Singularity software container technology, BIGwas performs resource-efficient and reproducible analyses on a local computer or any high performance compute system (HPC) with just one command, with no need to manually install a software execution environment or various software packages. For a single-command GWAS analysis with 974,818 individuals and 92 million genetic markers, BIGwas takes around 16 days on a small HPC system with only seven compute nodes to perform a complete GWAS QC and association analysis protocol. Our dynamic parallelization approach enables shorter runtimes for large HPCs.
Conclusions Researchers without extensive bioinformatics knowledge and with few computer resources can use BIGwas to perform multi-cohort GWAS with one million GWAS samples and, if desired, use it to build their own (genome-wide) PheWAS resource. BIGwas is freely available for download from http://github.com/ikmb/gwas-qc and http://github.com/ikmb/gwas-assoc.

Key words: GWAS; PheWAS; biobank; quality control; association testing; pipeline; Nextflow; Singularity; scalability

Introduction

Genome-wide association studies (GWAS), in which millions of genetic variants are tested across the genomes of study individuals to identify genotype-phenotype associations, have revolutionized the field of complex disease genetics over the last ten years. Decreasing costs of genome-wide genotyping with SNP arrays allow routine collection of GWAS data from deeply phenotyped biobank population cohorts, where many clinical features can be assessed beyond disease status. For this reason, it is possible to conduct GWAS studies with over one million samples [1] using GWAS data from large biobanks

Compiled on: May 14, 2021.
Draft manuscript prepared by the author.

such as the UK Biobank (UKB) [2] or the US Million Veterans Program [3]. This further allows phenome-wide association studies (PheWAS) to be conducted to systematically study the effects of one or many genetic variants over a wide range of human phenotypes. GWAS/PheWAS analyses with more than one million GWAS samples from dozens of biobanks enable, for example, rapid screening for target genes for therapeutic interventions for COVID-19 patients in the current pandemic (https://www.covid19hg.org/). However, while efficient software pipelines, such as PheWeb [4], for visualization, navigation and sharing of results of large-scale GWAS and PheWAS projects have been developed recently, there are still no easily executable and reproducible software pipelines available for performing large-scale quality control (QC) and association tests on a large number of individual (large-scale) GWAS or PheWAS data sets with one million GWAS samples. In particular, parallel and consistent processing and analysis of dozens of individual GWAS cohorts in the context of a mega-GWAS analysis is enormously time-consuming and can quickly become unmanageable if processed manually.

For basic GWAS QC and association analysis, software programs such as PLINK2 [5] can efficiently process and analyse whole-genome genotype data. Due to its broad functionality and efficient binary file format, PLINK2 is commonly used, along with self-written R and Bash scripts for visualization and further processing of files and results. To facilitate the execution and chaining of numerous PLINK commands and R programs, several data processing packages for GWAS analysis have been developed in R [6, 7] or other scripting languages [8]. In addition, software environments such as RICOPILI [9] or Odyssey [10] have been developed with advanced online manuals that bundle many Unix, Awk, Perl and/or PLINK2 commands for QC and association analysis. However, familiarization with these environments is usually time-consuming and is complicated by the fact that the user must perform a large number of installation steps for the own working environment and/or modify and manually execute a collection of Bash and R scripts in specific directories. Odyssey, for example, supports the use of software containers but lacks HPC support and thus the scalability for large GWAS data sets. Recently, the portable and reproducible GWAS QC and association testing software pipeline H3Agwas (currently version 3) has been developed by Baichoo and colleagues from the Pan-African community project H3ABioNet [11] that takes the input and output data from various QC and association testing automatically through several computationally-intensive processing steps. These workflows have been generated for processing small GWAS data sets but were not recommended for processing data sets with more than 50,000 GWAS samples. To the best of our knowledge, there is no portable (i.e. container-based) QC and association software pipeline that (i) can be executed with a single command without manual pre-installation, (ii) does not require reading and executing long online tutorials and (iii) can efficiently and quickly process one million GWAS samples as provided by large-scale GWAS/PheWAS biobank projects.

We developed BIGwas, a fast and efficient platform-independent software pipeline for QC and association testing of large-scale GWAS/PheWAS data sets, which automatically processes one million GWAS samples. By using Nextflow workflow technology [14] in combination with Singularity software container technology [15] (Methods), BIGwas can be started with a single command (Figure 1), with no need to manually install a full software execution environment or various software packages. BIGwas implements a variety of best practice

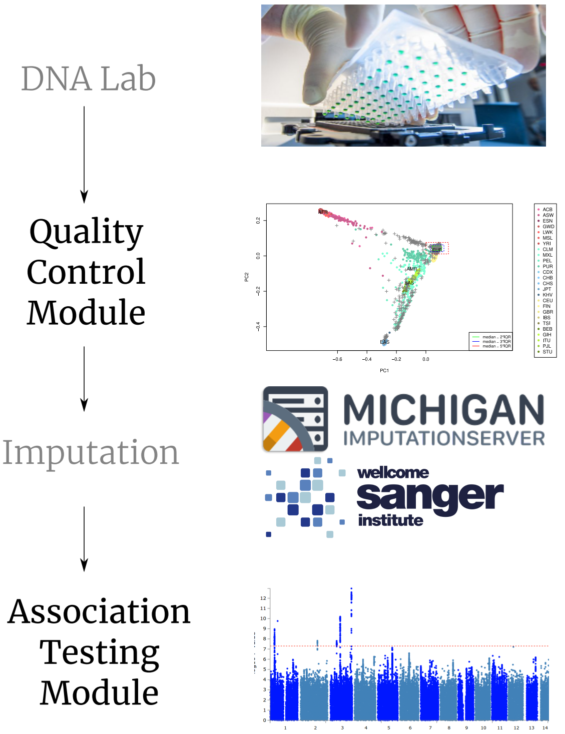

Figure 1. Overview of the single-command quality control and association testing workflow of BIGwas. Multiple GWAS data sets in PLINK format from any genotyping platform can be used as input for the Quality Control (QC) Module. Both the Quality Control Module and the Association Testing Module can be started and executed with a single command. The genotype output files of Quality Control Module in VCF format can be used directly as input for genotype imputation programs such as the TOPMed Imputation Server [12]. The VCF output files of the imputation process can be used directly as input for the Association Testing Module, which implements logistic/linear regression and mixed model association testing using PLINK [5] and SAIGE [13].

QC and GWAS mega-analysis protocols (see Methods) developed by the Inflammatory Bowel Disease Genetics Consortium (IIBDGC) [16, 17, 18] and The Severe Covid-19 GWAS Group [19]. The development of our container-based and reproducible biobank-scale GWAS/PheWAS software pipeline was further motivated by current large-scale GWAS and PheWAS screening projects, such as the UKB GWAS/PheWAS project of Benjamine Neale and colleagues (http://www.nealelab.is/uk-biobank/) or the Michigan Genomics PheWeb Initiative (http://pheweb.sph.umich.edu/about) in which GWAS analyses are performed for thousands of human traits with large GWAS data sets from UKB and the Michigan Genomics Initiative (MGI). The current PheWeb instance of the MGI displays UKB summary statistics of 28 million genetic markers assessed across 1,403 binary traits for 408,961 white British participants [20]. In these projects, the underlying QC and GWAS/PheWAS association analyses for thousands of traits run on large HPCs for several months or even years and are performed by large scientific working groups under great expenditure of time and personnel. To create a (genome-wide) PheWAS resource of this type, BIGwas can be used alongside the QC and mega GWAS analysis capability to test for association with a wide variety of traits in parallel or sequentially (requiring only one single program call per trait on a HPC) when numerous different phenotype information is available for the same individuals.

BIGwas comprises two different modules, namely the Quality Control (QC) Module and the Association Testing Module, that are part of a typical GWAS workflow (Figure 1). While the

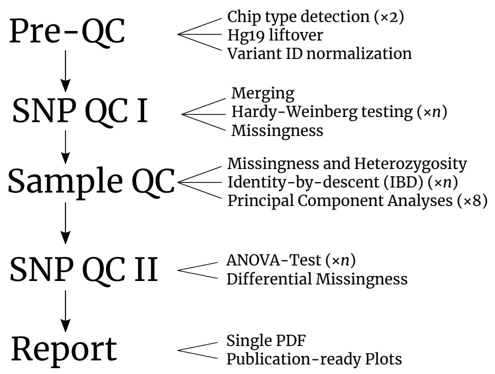

Figure 2. Workflow graph of the Quality Control (QC) Module of BIGwas, with the default number of threads used in each process (for details see Methods). *n* denotes that the number of parallel jobs or threads automatically scales with the number of samples in the data set.

QC module uses user-supplied GWAS data sets in the widely-adopted PLINK binary format, the Association Testing Module also allows input formats such as VCF (variant call format) that are usually delivered by genotype imputation servers, as with the TOPMed Imputation Server [12]. We compared the performance of BIGwas with the H3Agwas Pipeline Version 3 in terms of runtime and functionality, since H3Agwas is currently the only portable pipeline for reproducible GWAS that can be installed from a single source and started with just a few commands. To perform the benchmarks, we used both small (up to 20,000 GWAS samples) as well as large GWAS data sets (up to one million GWAS samples), including the current genotyped and imputed GWAS releases of UKB (September 2020) for COVID-19 case-control association analysis.

Methods

Quality Control Module

The Quality Control module works for binary as well as quantitative traits and is subdivided into five distinct phases: Pre-QC, SNP QC I, Sample QC, SNP QC II and Final Analysis and Report Generation. For a quality control based on quantitative traits, in the PLINK input Fam file, only the 6th column must be specified as a quantitative trait (for more information, see also http://zzz.bwh.harvard.edu/plink/data.shtml. In Pre-QC, an unlimited number of input GWAS data sets (batches) in PLINK format from different Affymetrix and Illumina platforms are, among other steps, matched with known variant databases from dbSNP and currently 140 Illumina/Affymetrix GWAS platforms and then brought to the same marker content. SNP QC I primarily filters variants based on missingness and Hardy-Weinberg equilibrium (in controls only) across and within batches (i.e. genotype files or self-defined batches) as well as batches from a particular phenotype and the entire control group with a user-defined false discovery threshold (FDR), allowing for differences in sample size between batches. The Sample QC removes samples based, among others, on missingness, increased or decreased heterozygosity rates, duplicate/relatedness testing (identity-by-state (IBS) and identity-by-descent (IBD) estimation) and population structure analysis/Tracy-Widom statistics, with or without projection on reference samples from HapMap and the 1000 Genomes Project. SNP QC II filtering steps include testing for differential missingness between phenotypes and controls, monomorphic sites, and significantly different allele frequencies of variants across the batches from a particular phenotype or the control group while correcting for population structure or ancestry via analysis of variance (ANOVA), with a user-defined FDR threshold. Finally, all information gathered throughout the QC pipeline is written to a single PDF report (see Figure 2). In principle, the QC module can be used for any European and non-European population. However, for the special case of an intended trans-ancestry GWAS study (for example to analyze GWAS samples of European, Asian and African descent), we recommend that a separate quality control be performed for each ethnic group.

Pre-QC

In large-scale consortium-based genome-wide association studies (GWAS) with dozens of GWAS input data sets collected from different gentoyping centers it is useful to apply SNP and sample QC metrics across and within genotyping batches for the identification of potential batch effects. For this task and the subsequent merging of data sets from same genotyping platforms, all genetic variants are annotated in the same way. This includes the mapping to a genome build, the naming of variants as provided by the dbSNP databases and the strand alignment. The Pre-QC phase brings all input data sets to a common annotation that is suitable for merging.

Individual Annotations

A batch affiliation can be defined independently from a single GWAS batch using the annotations file. In this file, separate batch identifiers can be assigned for the genotyping batch, country of origin and diagnosis (if non-control). Each of these identifiers will be used in principal component plots, histograms and ANOVA tests to identify batch effects within input data sets.

Assignment of variants to Pseudo-autosomal Regions

The first step consists of checking and assigning variants to the pseudoautosomal regions (PARs, which are inherited just like autosomal chromosomes) on chromosomes X and Y, as defined by genomic positions in the current genome builds. The genomic annotation of the PAR region have undergone a series of changes throughout different PLINK versions. In earlier versions, PLINK stored both PAR1 and PAR2 variants separate from the X (23) and Y (24) chromosomes, namely in a virtual chromosome 25, while newer PLINK versions use alpha-numerical chromosome codes such as `PAR1` and `PAR2`. As not every tool is aware of these changes, they are often mixed up in chromosomes 23, 24, 25, or all of them. The Pre-QC phase consistently moves both PAR1 and PAR2 variants back into chromosome 25 according to the coordinates published by the Genome Reference Consortium for GRCh37 [21]; after the QC, chromosome 25 variants are moved back to chr23 for the generation of the separate imputation vcf input files, since this is required by the TOPMed Imputation Server, see section on Variant Call Format Output below. If a data set presents heterozygous calls for males outside the PAR1/PAR2 regions, and hence, belong to the non-PAR regions on chromosomes X or Y, heterozygous genotype calls for males are likely to be sequencing errors and will be set to *missing*. The QC Module does not perform a check between self-reported and genetically determined biological sex based on chromosome X PLINK genotypes; we recommend using normalized signal intensities of SNPs on chromosome X as well as Y for this purpose.

Automatic Chip Type and Variant Annotation Detection

Since many GWAS platforms (microarray or chip type) have specific properties and exhibit potential biases, it is crucial for the QC to know the exact chip type from which the data set was generated. This step helps identifying the chip type by

matching chromosome IDs, variant IDs, base-pair positions and alleles of variants from PLINK's bim files against a set of currently 140 original GWAS platform content information files (called strand files) provided by Illumina and Affymetrix [22]. The results of the chip detection step are expressed as match rates (in percentages) and can not only be used to verify the true chip type, but also whether variant identifiers and/or the string information have already been manipulated by the user. Our current chip detection database contains 140 different chip types (including genome builds 37, 38 and partially 36) resulting in 415 chip versions that can be detected. The strand files are processed by a configurable number of threads (per default 2 threads only) where each thread processes a strand file as follows: First, every file entry (i.e. variant) of PLINK's bim file is matched against the strand file using variant ID, positition and alleles. After the final variant match, a composite match rate is calculated to assess how well a particular strand file (or chip type) matches the annotation in the bim file. After each string file is processed, the list of chip matches is sorted by a weighting formula, with m denoting the respective match rate:

$$m_{\text{sort}} = m_{\text{ID}} \cdot m_{\text{pos}} \cdot \max\left\{m_{\text{orig\_strand}}, m_{\text{plus\_strand}}\right\}$$

It is often the case, that variants are converted to the plus strand before QC. To make the match sorting robust, both the allele match rate for the original strand definitions and the allele match rate for the plus strand will be calculated. The strand files are then sorted by m_{sort} and can be used later for an optional genome build lift-over process (non-hg19 data and liftover to hg19). If the bim files match the plus strand better than the original strand information, a note is displayed in the PDF report. For performance reasons, the detection software has been written in the Rust programming language and is also available as a separate application (see Sect. Availability of Source Code and Requirements).

Automatic Genome Build Lift-over of Genomic Coordinates
Currently, the QC pipeline only supports data on genome build 19 (or GRCh37). If input data is in a different format, e.g. hg18 (GRCh36) or hg38 (GRCh38), the lift-over step can be explicitly enabled to perform an automatic lift-over to hg19. If the chip type is known beforehand or from the automatic chip type detection step (see above), a lift-over request can be set in the data set configuration file. A genome build conversion is often accompanied by the loss of some variants when variants in the target build are split into two or more variants or merged into one. This may cause unmappable or duplicate variants that will subsequently be removed from the data set. A warning will be displayed in the PDF report if a lift-over is requested and more than a pre-defined number of variants are lost due to this step (defaults to 10 %).

Matching of Variant Identifiers with External Databases
Different GWAS platforms often use different variant identifiers for the same variants. In this step, variant identifiers from PLINK's bim files are matched against a variant database containing identifiers of the Haplotype Reference Consortium (HRC) 1.1 reference panel [23], the UK10k cohort [24], the 1000 Genomes Phase 3 Project [25] and the db150 variant database [26]. If a single unique match is found, the respective variant name is retrieved and the original name replaced. In case the variant alleles match the strand complement of the reported alleles in the database, the variant is scheduled for strand alignment in a later step. Some genomic positions are covered by more than one variant, i.e. deletions that span multiple positions and overlap with SNPs, so that it is not clear which variant is to be assigned. In these cases, the name is set to the chr:pos format, e.g. `1:554127`. If no match could be found in the database, the original variant name is retained and prefixed with `unk_` to indicate that neither name, position, alleles nor strand alignment could be verified, which is often the case if the content is custom chip content. Variants with different names but same position and same alleles are classified as duplicates and one of the duplicates is discarded afterwards.

SNP QC I

During SNP QC I, the prepared data sets, which were preprocessed in parallel in the pre-QC phase, are merged into a single data set and filtered by missingness and Hardy-Weinberg equilibrium (in controls only) across all batches as well as single batches from a particular phenotype and the control group. DeFinetti diagrams [27] are created before and after SNP QC I to verify the quality of variants visually.

Merge Step
If several input data sets have been defined, they are merged into a single PLINK data set. However, the samples will still be analyzed taking into account the previously defined batches.

Missingness
Variants with a high genotyping error rate or which are only present in a subset of the data sets may now be discarded based on variant missingness. Variant missingness is calculated across all batches as well as single batches. The default missing call rate thresholds are 0.1 and 0.02, respectively. If desired, these can be reconfigured by the user.

Tests for Hardy-Weinberg equilibrium
Hardy-Weinberg equilibrium (HWE) tests are performed for controls only in a case-control study (and for all individuals if a quantitative trait is to be considered). HWE tests are performed for all variants from the autosomes (chromosomes 1-22) and the pseudo-autosomal regions (PAR). For women, the complete chromosome X is also tested, if available. Per default, variants deviating from HWE (with an FDR threshold of 10^{-5} in controls per default) across the entire batch collection with at most one batch being removed or falling below the FDR threshold in two single control batches are excluded. Batch-wise p-values are reported to find variants that fail HWE, among others, in a single control batch (but not in other control batches), in two or more control batches and in the whole collection of controls. Since this version of HWE testing is a computationally intensive task, the data set is divided into small pieces of 1000 variants each, which are then processed in parallel if the surrounding compute environment allows it (e.g. on an HPC cluster). Diagrams are also generated showing how many variants are removed in each specific case; if desired, the user can adjust the threshold parameters using these diagrams.

Sample QC

After SNP QC I, several sample QC measures will be calculated to identify samples showing a high variant missingness rate, an increased or decreased heterozygosity rate, to identify duplicate and related samples and to examine potential population substructure or batch effects by means of principal component analysis (PCA) and Tracy-Widom statistics, with reference samples from HapMap and the 1000 Genomes Project.

Individual Missingness and Heterozygosity
In this step, a list of sample outliers is generated through a series of computations that determine the sample missingness of each data set and the sample-wise heterozygosity rates across

all variants. For the former characteristic, the PLINK tool is run to generate a summary statistic of the genotyping rates per individual sample. Per default, samples that have a missing rate of $\geq$0.02 are removed. If desired, the user can adjust this threshold. The heterozygosity is determined with a second PLINK call, comparing the expected homozygosity rate with the observed homozygosity rate. Samples with a heterozygosity rate of $\pm 5\times$ standard deviation are classified as outliers. A scatter plot of the proportion called missing (x-axis) against the proportion of SNPs called heterozygote (y-axis) for each individual in the study is generated.

Controlling for population stratification and other confounders

The most common method for identifying (and subsequently removing) individuals with large-scale differences in ancestry is principal component analysis (PCA). To resolve within-Europe relationships (the default) and to test for population stratification, all samples are projected onto principal component (PC) axes generated from four intercontinental HapMap populations as well as 26 reference populations from the 1000 Genomes Project using FlashPCA [28]. The distribution of cases and controls are displayed along the first ten principal components stratified by phenotype. Individual GWAS samples (grey crosses) with values smaller than the median plus/minus five times the interquartile range (median ±5×IQR; samples outside the red square in PCA plot) represent PCA outliers for the first two PCs and are automatically removed (the default; adjustable). All remaining samples (without PCA outliers) are projected onto PS axes generated from 26 reference populations from the 1000 Genomes Project again. Samples are also visualized without reference population samples using FlashPCA, with samples colored by batch code, and the distribution of cases and controls are displayed along the first ten principal components stratified by phenotype. Tracy-Widom statistics [29] are further computed with EIGENSTRAT [30] to evaluate the statistical significance of each PC identified by PCA and to decide which top axes of variation are significant and should be used as covariates in association analyses. For each PCA, a set of independent (MAF >0.05) SNPs is calculated, excluding non-autosomes, variants in LD (leaving no pairs with r^2>0.2, within 50 kb windows) or within the extended major histocompatibility complex (xMHC; chr6:25-34Mb), and 11 high-LD regions as described by Price et. al. [31]. PCA plots with and without A/T and G/C SNPs are created to reveal potential undetected strand problems.

Super population codes from the 1000 Genomes Project are: AFR, African; AMR, Ad Mixed American, EAS, East Asian, EUR, European; SAS, South Asian; CHB, Han Chinese in Bejing, China; JPT, Japanese in Tokyo, Japan; CHS, Southern Han Chinese; CDX, Chinese Dai in Xishuangbanna, China; KHV, Kinh in Ho Chi Minh City, Vietnam; CEU, Utah Residents (CEPH) with Northern and Western Ancestry; TSI, Toscani in Italia; FIN, Finnish in Finland; GBR, British in England and Scotland; IBS, Iberian Population in Spain; YRI, Yoruba in Ibadan, Nigeria; LWK, Luhya in Webuye, Kenya; GWD, Gambia; MSL, Mende in Sierra Leone; ESN, Esan in Nigeria; ASW, Americans of African Ancestryin SW USA; ACB, African Caribbeans in Barbados; MXL, Mexican Ancestry from Los Angeles USA; PUR, Puerto Ricans from Puerto Rico; CLM, Colombians from Medellin, Colombia; PEL, Peruvians from Lima, Peru; GIH, Gujarati Indian from Houston, Texas; PJL, Punjabi from Lahore, Pakistan; BEB, Bengali from Bangladesh; STU, Sri Lankan Tamil from the UK; ITU, Indian Telugu from the UK.

Duplicate/Relatedness Detection

For robust duplicate/relatedness testing (identity-by-state (IBS) and identity-by-descent (IBD) estimation), we use the same set of independent variants, which was dynamically determined for PCA analysis (see above). Pairwise percentage IBD values were computed from a pruned subset of independent SNPs (see above) using PLINK. By definition, $P(IBD = 0) + P(IBD = 1) + P(IBD = 2) = 1$ and $\hat{\pi} := P(IBD = 2) + 0.5 \cdot P(IBD = 1)$ (proportion IBD). The expectation is that $IBD = 1$ for duplicates or monozygotic twins, $IBD = 0.5$ for first-degree relatives, $IBD = 0.25$ for second-degree relatives and $IBD = 0.125$ for third-degree relatives. Owing to genotyping error, LD and population structure, there is often some variation around these theoretical values and it is typical to remove one individual from each pair with an IBD value of > 0.1875, which is halfway between the expected IBD for third- and second-degree relatives. For these reasons an IBD value of > 0.98 identifies duplicates (adapted from [16]). One individual (the one showing greater missingness) from each pair with $\hat{\pi} > 0.1875$ is flagged in output files or is removed.

For kinship detection, by definition, every possible pair of samples is analyzed. The expected runtime is therefore quadratic with respect to the number of samples. With recent data sets growing past 500,000 samples, such as the UKB Data Release [2], this becomes a computationally intensive task, almost impossible to process on a single computer in an acceptable time frame. Our pipeline software automatically splits the workload and distributes the individual tasks to the available compute nodes in an HPC environment, if available, to process biobank-scale data. With the current, user-configurable scaling settings, and assuming that all split jobs can be run in parallel, the kinship detection would only take as much time as a 2,000-sample data set would take, even for large sample databases.

SNP QC II

After Sample QC, a few final SNP QC measures are calculated to identify variants where batches within a phenotype and the control group have different allele frequencies even after correction for potential population substructure and variants that show significantly different missingness between cases from the same phenotype and controls.

ANOVA Test

Significantly different allele frequencies of variants across the batches from a particular phenotype or the control group due to batch effects can be detected via analysis of variance (ANOVA) which adjusts for potential population structure from PCA analysis. This test for difference in the mean allele frequencies (after correcting for population stratification) across different batches from a particular disease or the control group. First, allele dosages are regressed against top PCs from PCA and residuals were obtained. P values are calculated from the ANOVA between the residuals of the genotypes and the batches. The null hypothesis is that all groups from a particular disease or from the group are simply random samples of the same population. Variants that have significantly different allele frequencies across the batches (with allowing a single batch to be removed) within phenotypic sets with an FDR threshold of 0.01 (the default) are removed. Robust detection of outlier variants is only possible with the availability of several different batches for the same diagnosis. Hence, this step is only performed if there is at least one defined diagnosis with at least five batches.

Differential Missingness

Given a case/control phenotype, the task is to detect platform/batch differences between case and control genotype data which may lead to false positive signals in association analysis. A Fisher's exact test on case/control missing call counts at each variant will be performed. Any variant which comes up as

highly significant under this test (P$\leq 10^{-15}$ per default) should be treated with great caution and will be excluded.

Final Analysis and Report Generation

In this stage, the final quality controlled (QCed) output has been generated as no more variants or samples will be removed based on quality metrics. To visualize the changes resulting from the removal of variant and sample outliers, a summary based on several PCA plots is generated.

Variant Call Format Output

The QCed GWAS data set is also converted to Variant Call Format (VCF) format so that it can be used as input for genotype imputation and association testing tools. Data sets are converted to the VCF 4.2 format [32] and split to chromosome-wise files as expected by many imputation tools such as the TOPMed Imputation Server [12].

Automatic Liftover to hg38 (optional)

The final VCF files are currently based on genome build hg19. Services such as the TOPMed imputation server [12], automatically convert VCF files to hg38, making it difficult to merge post-imputation data with pre-imputation data sets prepared in hg19. Optionally, the conversion to hg38 can be performed automatically by the pipeline using the UCSC liftOver Tools [33]. Due to licensing issues, the UCSC liftover tools [34] are not directly included in our implementation, but can be easily connected to our established UCSC liftover interface with a few steps. The same set of VCF files as for hg19 is generated in hg38 to facilitate the use of downstream imputation and association testing toolchains.

PDF Report Generation

Many other pipeline tools that provide GWAS QC include a number of program log files and other logs from which the number and IDs of removed variants and samples must be extracted for a final report. This can be a laborious and error-prone process. In our software, we introduce the automatic generation of a sophisticated PDF report that visualizes all changes and analysis of input data sets in plots and tables within a well-structured, single PDF document. A large collection of figures and tables before and after the QC are arranged side by side to allow easy verification of the results. Using the Nextflow engine, all process executions are further recorded in a single trace file. Our reporting software parses this trace file into an *execution tree*, where all generated files, such as log files, program output and graphics, can be accessed directly by their task name. A collection of custom parsers then uses the execution tree to create a template in the LaTeX typesetting language which is finally compiled into a PDF file. A working LaTeX installation is not required since all necessary software is included in the accompanied Singularity image.

Association Testing Module

Association analysis in GWAS/PheWAS is used to identify variants and regions of the genome associated with the phenotype of interest or to examine variants of special interest for their impact on a large collection of phenotypes. The most used software tools for single-variant association analysis are PLINK [5] and SAIGE [13], among others. Logistic/linear regression as implemented in PLINK is the de-facto gold standard for association analysis, with logistic/linear mixed models as implemented in SAIGE now being the new standard for biobank-scale GWAS and PheWAS. The Association Testing Module allows association analysis for binary and quantitative traits with

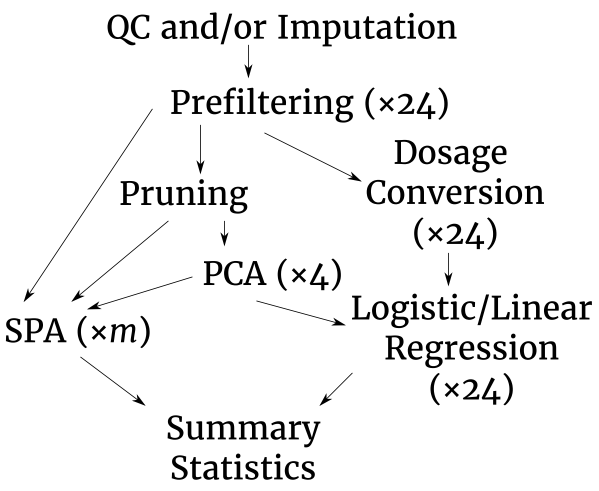

Figure 3. Workflow graph of the Association Testing Module of BIGwas, with default number of threads used in each process (for details see Methods). For example, association analysis is parallelized over a fixed number of chromosomes ($\times 24$). m denotes that the number of threads linearly scales with the number variants. SPA: saddle point approximation to calibrate unbalanced case-control ratios (see Methods). PCA: principal component analysis.

PLINK and SAIGE on both imputed and non-imputed GWAS data sets on different genome builds, both with and without covariates, including chromosome X analysis, combined in a single-command. For quantitative traits, the user has to set the parameter `-trait` to "quantitative" instead of "binary" (the default). In this case, PLINK as well as SAIGE will then automatically perform an association analysis for a quantitative trait. All processes, including conversion steps, are largely processed in parallel, so that each GWAS association analysis for each phenotype can be accomplished by a single run (see Figure 3).

Data Preparation

No further genotype data preparation step is required if the input data set, a set of chromosome-wise VCF files (optionally including chromsome X), is obtained either from the Quality Control Module or from genotype imputation programs such as the TOPMed Imputation Server and contains dosage and/or genotype information. VCF files must have a DS tag for dosage data or a GT tag for genotyped data. If both are present, DS is chosen.

Sample Selection, Covariate Preparation and Null Model Building for SAIGE

To select a desired subset of samples for association analysis, an optional PLINK FAM file to update sex information and case-control status can be specified, and an optional covariate file if covariate adjustment is desired. In this case only the joint set of samples from the sample list of the VCF and the optional FAM file (and the optional covariate file) is used for model building (in SAIGE) and association analysis; no further modification of the VCF input files is necessary. For model building in SAIGE using saddle point approximation (SPA) [13] in order to calibrate unbalanced case-control ratios, the null logistic/linear mixed model is fitted based on a set of independent variants (see section Controlling for population and other confounders, Methods), with all independent variants also having an imputation quality score of $r^2 \geq 0.8$ (threshold configurable). With the same set of independent variants PCA analysis is performed using FlashPCA2 [28] to calculate top PCs (default n=10) to ad-

just for potential population stratification.

PLINK Association Testing

PLINK's logistic/linear regression association module requires a custom text-based file with genotype dosage information (genotype probability format) which is generated on-the-fly from imputed or non-imputed VCF input files. Genotype dosage-based association analysis is conducted with the top PCs (default n=10) from PCA and user-defined optional covariates from the covariate file. For chromosome X association analysis, haplotypic allele calls outside the PAR regions in males are converted to homozygous calls by doubling the haplotypic allele (assuming inactivation of large parts of one of the two female X chromosomes [35]), and sex is used as a covariate for association testing of the non-PAR regions on chromosome X.

SAIGE Association Testing

One of the main features of SAIGE is its ability to perform single-marker association testing for extremely unbalanced case-control ratios and rare variants [13]. While SAIGE's computational load is comparable to logistic or linear regression as performed by PLINK, SAIGE can directly read compressed VCF files typically generated from genotype imputation programs, including imputed dosage data and genotype-only calls, and further supports the memory efficient BGEN format as used in the UK Biobank imputed data releases. SAIGE allows much finer control on parallelization by allowing the association analysis on specified genomic parts of a VCF/BGEN without the need to cut the file into smaller chunks. This technique does not only save runtime that would otherwise be consumed by splitting up VCF files but also large amounts of temporary file storage and I/O load. As no extra computational load is required for splitting of chromosomal files, the scalability of how many jobs can be executed in parallel is only limited by the available computational resources, and the overhead is very low with respect to the chunk size of the genomic parts. Using only a sub-sample of all samples from VCF input files is not supported by SAIGE yet. For this reason, we have written a routine that automatically extracts a sub-sample from the VCF files based on a user-defined PLINK FAM file before starting the analysis with SAIGE. Association analysis with SAIGE (mixed effect model + covariates) is conducted using the top PCs (default n=10) from PCA and user-defined optional covariates from the covariate file. The same chromsome X analysis method used by PLINK is performed in SAIGE.

Compatibility with UK Biobank Data

The UKB's latest GWAS data release contains, in addition to unimputed genotype data in PLINK format, pre-imputed data in the BGEN format instead of the much more common VCF format. However, most computational procedures of the Association Testing Module, especially the conversion routines that converts VCF files to PLINK's genotype dosage format, heavily rely on the VCF format specification. BGEN, while much more efficient than VCF, is a binary format that is not designed to be processed by scripts, and as such, cannot directly be processed into PLINK's genotype dosage format. Since the more common VCF format is a generic format used by biobanks and genotype imputation programs (TOPMed and Sanger Imputation server [12], among others) worldwide, we chose to drop BGEN support and therefore, in addition to SAIGE, can still keep PLINK as a de-facto gold standard tool for regression-based association analysis in our association testing workflow.

However, we included a set of scripts to perform SAIGE association testing on UKB BGEN files. This "pipeline" is a feature-reduced version that only performs the absolutely necessary steps to run SAIGE on the input data but uses the same fine-grained parallelization techniques as used in the SAIGE/Plink VCF-based pipeline. External covariates, such as principal components, sex and age, can be extracted from UKB sample information databases and supplied to the association testing process.

Implementation Details

We use the free and open-source Nextflow [14] pipeline software for easy parallelization of processes, HPC-based development and pipeline control. Singularity, a container format and software focused on scientific computation and reproducibility, is used to provide a single-command experience without the need to install an entire software execution environment of different software packages.

Nextflow

While both the QC Control Module and the Association Testing Module are on the same level of runtime performance as comparable GWAS pipelines that analyse a smaller number of samples and variants (compare Tables 2 and 3), the real challenge of existing GWAS pipelines becomes apparent with larger data sets. For example, many QC and association testing tools use the duplicate/relatedness detection feature (see Methods) to remove related individuals or duplicate samples from the data set. As every sample is tested against every other sample, the total runtime grows quadratic in relation to the number of samples, i.e. the time complexity is $\mathcal{O}(n^2)$.

Nextflow, a tool specifically designed to run bioinformatics applications, therefore provides a suitable environment to facilitate the parallelism of processes in all workflows. It extends the Unix pipes model with a fluent data descrition language (DSL), allowing you to handle complex stream interactions easily. In our case, we use a dynamic job creation approach to automatically determine the number of parallel jobs required to perform an operation within a reasonable time frame. For example, in the duplicate/relatedness detection step, we use both PLINK's `-parallel` mode and Nextflow's job splitting feature to achieve fast processing of large input files. The same mechanism is also used for other computationally intensive operations, such as our "leave-one-batch-out Hardy-Weinberg test" or the SAIGE association testing procedure. Nextflow takes care of the creation, workspace separation and merging of all jobs, avoids user-defined error-prone synchronization of all jobs, and the merging of results from complex analysis pipelines. All the intermediate results produced during the pipeline execution are automatically tracked.

Another major advantage of Nextflow is the already integrated handling and agnostic use of HPC cluster resources (i.e. it is able to work under different environments). With Nextflow, it is possible to write parallel and concurrent pipeline workflows without having knowledge (and thus knowing all dependencies) of a specific cluster environment, such as SGE, SLURM or PBS/TORQUE. This makes it possible to provide pipelines for a wide range of HPC and non-HPC users. The deep integration of different storage solutions like ECS object storage or S3 buckets and container tools such as Docker and Singularity make Nextflow a first choice for a portable, widely available platform, with the only requirement of a working Java installation.

Singularity

Our general Nextflow script code base is hosted on GitHub. However, the included pipeline scripts make heavy use of external tools, such as the well-known PLINK sofware [5] for GWAS data. Since each workflow is usually created with specific versions of tools in mind, it is often the case that developers need to install multiple versions of the same software and must take special care in managing them. Conda [36] and Lmod [37] assist in setting up the respective software environments, but the required tools must still be installed manually by the user.

In our case we decided to prepare the execution environment, including an entire operating system, in a publicly-hosted container image, including all specific program versions. This not only eliminates the burden and error-proneness of manually creating a new environment, but also ensures that each user uses exactly the same program versions and therefore can reproduce any result with the same input, without any problems with deployment on different operating systems, user permissions or configuration management.

Another advantage of using an entire file system is not only the possibility to add programs as desired, but to include any file. We use these capabilities to provide ready-to-use Hapmap2 [38] and 1000 Genomes [25] data sets that serve as reference samples, for example, for principal component analysis (PCA), as well as a comprehensive but slim database of NCBI Reference SNP ID numbers [39].

Results

Benchmark Settings

In Table 1 we compare the features of the QC Module of BIGwas (for details see Methods) with the features of the QC module from the H3Agwas Pipeline Version 3, a portable and reproducible GWAS QC and association test software workflow developed by the pan-African community project H3ABioNet (see Introduction). The H3A QC module was developed to perform a robust and efficient QC workflow for over 30,000 DNA samples genotyped using a custom designed African genotyping array as part of H3ABioNet [11]. Since many similar features were implemented in both software pipelines (although there is no explicit support for multiple GWAS data sets (i.e. batches) in H3A QC), the runtime performance can be compared very well in a benchmark. We have not made a feature comparison for the association pipelines of BIGwas (for details see Methods) and H3A, since the authors of the H3A Association Pipeline have made it clear that the purpose of the H3A Association Pipeline is to perform a very basic logistic/linear regression association analysis with PLINK (https://github.com/h3abionet/h3agwas/). The use of linear mixed models for association testing is further supported, but only for genotypes in PLINK format (and not imputed data in VCF format as delivered by genotype imputation servers) and without support for pre- or post-processing of input and output files. For this reason, we only compared the runtime of logistic/linear regression and mixed model testing with BIGwas (PLINK and SAIGE) versus logistic/linear regression with H3A (PLINK).

We conducted benchmark tests on our HPC system with 68 computational nodes and 1,336 CPU cores in total, which enables highly parallel computing. However, since in a multi-user HPC system not all resources are usually available to a single user, and in order to demonstrate that BIGwas uses resources economically, for benchmarking we allowed submitting a maximum of 150 jobs in parallel (corresponds to 7 compute nodes; configurable by the user) for testing both the QC Module and the Association Testing Module of BIGwas. For benchmarking H3A, we made no restrictions on HPC resources (thus allowing 1,336 jobs in parallel) to test the maximum performance of H3A. For each benchmark data set consisting of a different number of GWAS samples and genetic variants (Table 2 and Table 3), we used Nextflow's built-in reporting function to determine the elapsed wall-clock time. Each run-time analysis was performed three times on an empty HPC cluster to account for potential fluctuations in HPC cluster performance.

Table 1. Feature comparison between the QC Module of BIGwas (for details see Methods) and the H3A QC module of the H3Agwas Pipeline Version 3 [11]. The QC Module of BIGwas performs, among other things, an automatic upscaling of job parallelization with respect to the number of samples and variants and allows processing an unlimited number of input GWAS data sets (i.e. batches) with a single command.

Feature	BIGwas QC	H3A QC
Duplicate Removal	✓	✓
RsID dbSNP Assignment	✓	✗
Hardy-Weinberg Equil. (HWE) Test	✓	✓
HWE Test (batch-wise leave-one-out)	✓	✗
IBD/Relatedness Check	✓	✓
Missingness Check	✓	✓
Heterozygosity Check	✓	✓
PCA Outlier Detection	✓	✗[1]
Low-MAF Removal	✗	✓
Pseudoautosomal Region (PAR) Check	✓	✗
VCF Output	✓	✓
Genome Build Liftover	optional	✗
Dynamic Parallelization of Jobs	✓	✗[2]
PDF Report	✓	✓

[1] Principal component analysis (PCA) is calculated but ancestry outliers are not determined
[2] Parallelization effort is manually configurable but does not automatically scale

Table 2. Runtime Metrics of the QC Module of BIGwas compared to the QC module of the H3Agwas Pipeline Version 3 [11] for several different-sized GWAS data sets. A GWAS input data set with 976,584 samples and 803,113 genetic variants (i.e. twice the size of the current UK Biobank GWAS data set) can be quality-controlled in less than 7 days with one command of the BIGwas software, whereby only 150 jobs (configurable; equivalent to about 7 compute nodes on our HPC cluster system) are used in parallel. (*) indicates that the runtime of H3A has exceeded the maximum time allocation of 10 days for the HPC system despite the possibility to use a maximum of 1,336 jobs in parallel.

Samples	Variants	BIGwas Runtime	H3A Runtime
200	500	6 min	4 min
500	1,000	6 min	7 min
1,000	5,000	6 min	9 min
5,000	50,000	8 min	18 min
5,000	250,000	15 min	35 min
10,000	250,000	15 min	57 min
20,554	700,078	2 h 15 min	8 h 57 min
488,292	231,151	3 d 1 h	*
488,292	803,113	4 d 22 h	*
976,584	803,113	6 d 12 h	*

Benchmark Results

Table 2 shows runtime benchmarks of the QC Module of BIGwas (for details see Methods) compared to the QC module of the H3Agwas Pipeline Version 3. For comparison with H3A runtimes, we set configurable parameters, such as missingness thresholds, to similar values where possible and used the recommended commands for the respective benchmark data sets. Data sets in the upper part were subsampled from in-house GWAS data sets gentoyped using Illumina's Global Screening (GSA) Array v1.0. In the lower part, unimputed Axiom Array data of 488,292 individuals of European ancestry from UKB was used (see Acknowledgements). The data set of 976,584 samples and 803,113 SNPs represents an artificially created GWAS data set (from the original COVID-19 UK Biobank unimputed GWAS data release, September 2020, with 1,485 COVID-19 cases and 486,807 controls), which was created by duplicating the number of samples from the UK Biobank data set.

The theoretical algorithmic runtime complexity of the QC is quadratic with respect to the number of samples and linear with the number of variants $\mathcal{O}(n^2 + m)$, since in identity-by-descent (IBD) analysis each sample is checked against every other sample both in the H3A as well as BIGwas. The IBD calculation becomes the most time-consuming part from 5,000 samples upwards in H3A QC and leads to a preliminary termination of H3A QC for the UKB data set (Table 2). For 5,000 GWAS samples upwards, our implementation begins with a dynamic parallelization process (see Methods), i.e. scheduling (at maximum 150) jobs (configurable) to distribute the workload across multiple compute nodes in order to reduce the runtime (not shown) with respect to the number of samples. The number of additional jobs scales with the number of samples to keep the runtime for IBD analysis low (see processes marked with $\times n$ in Figure 2), thus the runtime of BIGwas increases much slower than that of H3A. Algorithmic complexity in other time-consuming applications such as Hardy-Weinberg and ANOVA testing is generally linear in the number of variants but we found it useful to also scale the number of parallel jobs or threads automatically with the number of samples (also marked with $\times n$ in Figure 2). Our resource-efficient intra- and inter-node software implementation (see Methods) shows that BIGwas did not exceed the maximum number of 150 parallel jobs allowed by us in our benchmark, and that BIGwas is able to analyze arbitrarily large GWAS input data sets with one million GWAS samples. Despite the possibility to use a maximum of 1,336 parallel jobs, H3A exceeded a runtime of 10 days for the UKB data set with 488,292 samples and a reduced set of 231,151 (linkage disequilibrium pruned) variants (Table 2). Results of BIGwas are summarized in a pdf report (see Methods) and output files (on either genome build hg19 and/or hg38; configurable) can be used directly for association testing and/or as an input for genotype imputation services.

Table 3 shows runtime benchmark results of the Association Testing Module of BIGwas (for details see Methods) compared to the association module of H3A. Data sets in the upper part were subsampled from in-house GWAS data sets gentoyped using Illumina's Global Screening (GSA) Array v1.0. In the lower part, pre-imputed GSA data of 5,480 individuals as well as pre-imputed Axiom Array data of 487,409 individuals of European ancestry from UKB was used (see Acknowledgements). The data set of 974,818 samples and 92,775,302 SNPs represents an artificially created imputed GWAS data set (from the orginal COVID-19 UK Biobank imputed GWAS data release, September 2020, with 636 cases and 486,773 controls), which was created by duplicating the samples of the UKB data set. Association analysis included top 10 principal components from PCA, sex, age, sex*age and age*age as covariates.

Table 3. Runtime Metrics of the Association Testing Module of BIGwas (regression analysis with PLINK and mixed model analysis with SAIGE) compared to association testing module of the H3Agwas Pipeline Version 3 (regression analysis with PLINK) [11] for several different-sized GWAS data sets. An imputed genome-wide input data set with almost one million samples and more than 92 million genetic variants (i.e. twice the size of the imputed UKB GWAS data set) can be tested for association within 10 days with one command of the BIGwas software, whereby only 150 jobs (configurable; equivalent to about 7 compute nodes on our HPC cluster system) are used in parallel. (*) indicates that H3A uses PLINK genotype files as input (but not imputed allele dosages).

Samples	Variants	BIGwas Runtime	H3A Runtime
10,000	250,000	21 min	8 min
10,000	700,078	54 min	16 min
20,554	700,078	1 h 33 min	1 h 23 min
5,480	81,708,012	14 h 26 min	*
487,409	92,775,302	4 d 18 h	*
974,818	92,775,302	9 d 14 h	*

The theorethical runtime of BIGwas increases linear with the number of variants $\mathcal{O}(m)$. The runtimes shown in Table 3 were achieved with a maximum number of parallel jobs of 150 for BIGwas, and with no restrictions on the number of parallel jobs for H3A (at maximum 1,336 jobs in parallel). By default, our pipeline module schedules one job for 5,000 genetic variants (marked with $\times m$ in Figure 3) for the limited number of maximum 150 parallel jobs (configurable). Our resource-efficient intra- and inter-node software implementation (see Methods) enables BIGwas to analyze arbitrarily large GWAS input data sets with millions of GWAS samples and millions of imputed genetic variants. Although SAIGE and PLINK association testing in BIGwas is performed in parallel in one run of the Association Testing Module, the runtime of BIGwas is only slightly longer than that of H3A, which performs association testing with PLINK only, without pre- and post-processing of input and output files. Some new features have been implemented in the Association Testing Module of BIGwas, which are not covered by the original implementations of SAIGE and PLINK (see Methods): Before association tests are performed, an on-the-fly sample filtering is conducted to exclude certain samples (e.g. based on a sample exclusion list; see Methods) or variants (e.g. based on the imputation score threshold r^2; see Methods) from PLINK or VCF input data sets, without the need to manually remove them first, which is usually a labor-intensive step for the user. In addition, an automated principal component analysis (PCA; configurable) is integrated into SAIGE mixed-model association analysis, so that model fitting analysis and association testing (along with principal components from PCA and/or user provided covariates) are performed in SAIGE in a single step; association testing for chromosome X is also automated for PLINK and SAIGE (see Methods). Association summary statistics output files as well as best-guess genotype calls in PLINK format are produced for different genome builds (hg19 and/or hg38; configurable).

Discussion

We have introduced BIGwas, a portable (i.e. Singularity container-based), fully automated GWAS quality control (QC) and association pipeline in Nextflow that can be executed with a single command without manual installation by the user. BIGwas differs from existing GWAS data processing packages in R in that it allows standardized, reproducible, and high-throughput processing of GWAS data sets with a single

command, whereas existing GWAS R packages allow for more flexible processing, for example, with respect to the order of processing steps. The current UKB GWAS release with 487,409 GWAS samples can be analysed with BIGwas within 8 days (4 days for QC and 4 days for association testing) on a small HPC with 7 compute nodes. For a GWAS data set with up to one million individuals BIGwas needs on average 16 days on a HPC with 7 compute nodes. Using our dynamic parallelization approach, shorter runtimes can be achieved with a larger HPC. For quality control (QC) the theoretical runtime increases quadratically with the number of samples and linear with the number of variants $\mathcal{O}(n^2 + m)$. For association testing the runtime increases linear with the number of variants $\mathcal{O}(m)$. As sample sizes in large GWAS/PheWAS biobank studies, for example, for COVID-19 research (https://www.covid19hg.org/), have grown to millions of GWAS samples and GWAS data sets of this size are likely to be used more frequently in the future, the efficiency and ease of execution of BIGwas with a single command will become an important criterion for conducting GWAS/PheWAS studies.

To enable a reproducible analysis of one million GWAS samples on any common compute platform, we have combined various code optimizations of our existing GWAS software with Nextflow workflow and Singularity container technology. Our Nextflow pipelines are inherently parallel and can be transparently scaled up or down, and they provide an abstraction layer between the logic of the pipeline and the execution layer (i.e. they can be executed on multiple platforms without changing the source code). With the Singularity container technology, we have packed all our Nextflow workflows, software and libraries, as well as publicly-available reference data into a single Singularity container that runs on any standard computer platform without manual download or installation by the user.

BIGwas implements a complete GWAS analysis protocol which was developed for large-scale GWAS studies of the Inflammatory Bowel Disease Genetics Consortium (IIBDGC) and the Severe Covid-19 GWAS Group[17, 18, 19]. BIGwas is not yet suitable for GWAS analysis of family data or GWAS analysis across populations of worldwide ethnicities. To enable these analyses, extensions to our QC and the association testing pipelines or the use of alternative statistical methods may be required. In the future, we aim to test the usability of further genome-wide analysis approaches, such as gene-gene interaction tests, for use with Nextflow and Singularity technology.

Availability of source code and requirements

- Project name: IKMB GWAS QC and Association Testing Pipelines
- Project home page: http://github.com/ikmb/gwas-qc and http://github.com/ikmb/gwas-assoc
- Operating system: Linux
- Programming language: Nextflow, Bash, Python, Perl, R, Rust
- Other requirements: Java 8 or higher, Singularity 3.4 or higher
- Bio.tools identifier: https://bio.tools/bigwas_qc_pipeline and https://bio.tools/bigwas_assoc_pipeline
- SciCrunch.org Research Resource Identification Initiative ID (RRID): SCR_019240 and SCR_019241

This project makes use of several open source tools, namely PLINK 1.9 [40], PLINK 2 [5], bcftools 1.10 from the HTSlib project [41], FlashPCA 2.0 [28], EIGENSTRAT 4.2[30] and SAIGE 0.42.1[13]. For the availability of source code, we refer to the respective publications. Several more native binaries were created and included for performance reasons. Their source codes are available as follows:

- *chipmatch*, a tool to match Plink's BIM files against the Will Rayner's strand database [22]: https://github.com/ikmb/chipmatch
- *genericplotter*, a high-performance heat map plotting tool for almost arbitrary, very large data sets: https://github.com/ikmb/genericplotter

UK Biobank (UKB) COVID-19 GWAS data release (September 2020): https://biobank.ndph.ox.ac.uk/ukb/exinfo.cgi?src=COVID19

Availability of supporting data and materials

An example data set is included in the software packages, see project home page. The example data set is a subset of the 1000 Genomes Project, using 2504 samples with randomized sex and phenotype, and 50,000 randomly-selected variants.

Declarations

Ethics approval and consent to participate

All participants provided written informed consent, and the study was approved by the ethics boards of the participating institutions (see Acknowledgements) in agreement with the Declaration of Helsinki principles.

Consent for publication

Not applicable.

Competing Interests

The authors declare that they have no competing interests.

Author's Contributions

Jan Christian Kässens: Conceptualization, Methodology, Software, Benchmarks, Writing **Lars Wienbrandt:** Software, Benchmarks, Writing, Visualization **David Ellinghaus:** Conceptualization, Methodology, Software, Formal analysis, Data curation, Supervision, Writing

Acknowledgements

The authors would like to thank Andre Franke for helpful discussions regarding the evaluation of the UK Biobank data, Frauke Degenhardt for sharing R scripts for visualisation of results from principal component analysis and Florian Uellendahl-Werth and Mareike Wendorff for helpful discussions on R code optimization. Benchmarks and scalability tests have been conducted using the UK Biobank Resource (Project #61199, "Biobank-Scale Configurable Quality Control and Association Testing for GWAS"). This work was supported by the German Federal Ministry of Education and Research (BMBF) within the framework of the Computational Life Sciences funding concept (CompLS grant 031L0165) and iTREAT (grant 01ZX1902A). The study received infrastructure support from the DFG Cluster of Excellence 2167 "Precision Medicine

in Chronic Inflammation (PMI)" (DFG Grant: "EXC2167"), the DFG research unit "miTarget" (Projectnummer 426660215) and the PopGen Biobank (P2N 2016-101).

References

1. Evangelou E, Warren HR, Mosen-Ansorena D, Mifsud B, Pazoki R, Gao H, et al. Genetic analysis of over 1 million people identifies 535 new loci associated with blood pressure traits. Nature Genetics 2018 Sep;50(10):1412–1425. https://doi.org/10.1038/s41588-018-0205-x.
2. Sudlow C, Gallacher J, Allen N, Beral V, Burton P, Danesh J, et al. UK Biobank: An Open Access Resource for Identifying the Causes of a Wide Range of Complex Diseases of Middle and Old Age. PLOS Medicine 2015 Mar;12(3):e1001779. https://doi.org/10.1371/journal.pmed.1001779.
3. Klarin D, Damrauer SM, Cho K, Sun YV, Teslovich TM, Honerlaw J, et al. Genetics of blood lipids among ~300, 000 multi-ethnic participants of the Million Veteran Program. Nature Genetics 2018 Oct;50(11):1514–1523. https://doi.org/10.1038/s41588-018-0222-9.
4. Taliun SAG, VandeHaar P, Boughton AP, Welch RP, Taliun D, Schmidt EM, et al. Exploring and visualizing large-scale genetic associations by using PheWeb. Nature Genetics 2020 Jun;52(6):550–552. https://doi.org/10.1038/s41588-020-0622-5.
5. Chang CC, Chow CC, Tellier LC, Vattikuti S, Purcell SM, Lee JJ. Second-generation PLINK: rising to the challenge of larger and richer datasets. GigaScience 2015 Feb;4(1). https://doi.org/10.1186/s13742-015-0047-8.
6. Gogarten SM, Bhangale T, Conomos MP, Laurie CA, McHugh CP, Painter I, et al. GWASTools: an R/Bioconductor package for quality control and analysis of genome-wide association studies. Bioinformatics 2012 Oct;28(24):3329–3331. https://doi.org/10.1093/bioinformatics/bts610.
7. van der Most PJ, Vaez A, Prins BP, Munoz ML, Snieder H, Alizadeh BZ, et al. QCGWAS: A flexible R package for automated quality control of genome-wide association results. Bioinformatics 2014 Jan;30(8):1185–1186. https://doi.org/10.1093/bioinformatics/btt745.
8. Perreault LPL, Legault MA, Asselin G, Dubé MP. genipe: an automated genome-wide imputation pipeline with automatic reporting and statistical tools. Bioinformatics 2016 Aug;p. btw487. https://doi.org/10.1093/bioinformatics/btw487.
9. Lam M, Awasthi S, Watson HJ, Goldstein J, Panagiotaropoulou G, Trubetskoy V, et al. RICOPILI: Rapid Imputation for COnsortias PIpeLIne. Bioinformatics 2019 Aug;https://doi.org/10.1093/bioinformatics/btz633.
10. Eller RJ, Janga SC, Walsh S. Odyssey: a semi-automated pipeline for phasing, imputation, and analysis of genome-wide genetic data. BMC Bioinformatics 2019 Jun;20(1). https://doi.org/10.1186/s12859-019-2964-5.
11. Baichoo S, Souilmi Y, Panji S, Botha G, Meintjes A, Hazelhurst S, et al. Developing reproducible bioinformatics analysis workflows for heterogeneous computing environments to support African genomics. BMC Bioinformatics 2018 Nov;19(1). https://doi.org/10.1186/s12859-018-2446-1.
12. Das S, Forer L, Schönherr S, Sidore C, Locke AE, Kwong A, et al. Next-generation genotype imputation service and methods. Nature Genetics 2016 Aug;48(10):1284–1287. https://doi.org/10.1038/ng.3656.
13. Zhou W, Zhao Z, Nielsen JB, Fritsche LG, LeFaive J, Taliun SAG, et al. Scalable generalized linear mixed model for region-based association tests in large biobanks and cohorts. Nature Genetics 2020 May;52(6):634–639. https://doi.org/10.1038/s41588-020-0621-6.
14. Tommaso PD, Chatzou M, Floden EW, Barja PP, Palumbo E, Notredame C. Nextflow enables reproducible computational workflows. Nature Biotechnology 2017 Apr;35(4):316–319. https://doi.org/10.1038/nbt.3820.
15. Kurtzer GM, Sochat V, Bauer MW. Singularity: Scientific containers for mobility of compute. PLOS ONE 2017 May;12(5):e0177459. https://doi.org/10.1371/journal.pone.0177459.
16. Anderson CA, Pettersson FH, Clarke GM, Cardon LR, Morris AP, Zondervan KT. Data quality control in genetic case-control association studies. Nature Protocols 2010 Aug;5(9):1564–1573. https://doi.org/10.1038/nprot.2010.116.
17. Ellinghaus D, Jostins L, Spain SL, Cortes A, Bethune J, Han B, et al. Analysis of five chronic inflammatory diseases identifies 27 new associations and highlights disease-specific patterns at shared loci. Nature Genetics 2016 Mar;48(5):510–518. https://doi.org/10.1038/ng.3528.
18. Huang H, Fang M, Jostins L, Mirkov MU, Boucher G, Anderson CA, et al. Fine-mapping inflammatory bowel disease loci to single-variant resolution. Nature 2017 Jun;547(7662):173–178. https://doi.org/10.1038/nature22969.
19. Ellinghaus D, Degenhardt F, Bujanda L, Buti M, Albillos A, Invernizzi P, et al. Genomewide Association Study of Severe Covid-19 with Respiratory Failure. New England Journal of Medicine 2020 Oct;383(16):1522–1534. https://doi.org/10.1056/nejmoa2020283.
20. Zhou W, Nielsen JB, Fritsche LG, Dey R, Gabrielsen ME, Wolford BN, et al. Efficiently controlling for case-control imbalance and sample relatedness in large-scale genetic association studies. Nature Genetics 2018 Aug;50(9):1335–1341. https://doi.org/10.1038/s41588-018-0184-y.
21. Church DM, Schneider VA, Graves T, Auger K, Cunningham F, Bouk N, et al. Modernizing Reference Genome Assemblies. PLoS Biology 2011 Jul;9(7):e1001091. https://doi.org/10.1371/journal.pbio.1001091.
22. Rayner NW, McCarthy MI. Development and Use Of a Pipeline to Generate Strand and Position Information for Common Genotyping Chips; 2011, poster presented at the ASHG 2011, Montreal, Canada.
23. McCarthy S, Das S, Kretzschmar W, Delaneau O, Wood AR, Teumer A. A reference panel of 64, 976 haplotypes for genotype imputation. Nature Genetics 2016 Aug;48(10):1279–1283. https://doi.org/10.1038/ng.3643.
24. The UK10K Consortium. The UK10K project identifies rare variants in health and disease. Nature 2015 Sep;526(7571):82–90. https://doi.org/10.1038/nature14962.
25. The 1000 Genomes Project Consortium. An integrated map of genetic variation from 1, 092 human genomes. Nature 2012 Oct;491(7422):56–65. https://doi.org/10.1038/nature11632.
26. Sherry ST. dbSNP: the NCBI database of genetic variation. Nucleic Acids Research 2001 Jan;29(1):308–311. https://doi.org/10.1093/nar/29.1.308.
27. Goddard KAB, Ziegler A, Wellek S. Adapting the logical basis of tests for Hardy-Weinberg Equilibrium to the real needs of association studies in human and medical genetics. Genetic Epidemiology 2009 Nov;33(7):569–580. https://doi.org/10.1002/gepi.20409.
28. Abraham G, Qiu Y, Inouye M. FlashPCA2: principal component analysis of Biobank-scale genotype datasets. Bioinformatics 2017 May;33(17):2776–2778. https://doi.org/10.1093/bioinformatics/btx299.
29. Patterson N, Price AL, Reich D. Population Structure and

Eigenanalysis. PLoS Genetics 2006;2(12):e190. https://doi.org/10.1371/journal.pgen.0020190.
30. Price AL, Patterson NJ, Plenge RM, Weinblatt ME, Shadick NA, Reich D. Principal components analysis corrects for stratification in genome-wide association studies. Nature Genetics 2006 Jul;38(8):904–909. https://doi.org/10.1038/ng1847.
31. Price AL, Weale ME, Patterson N, Myers SR, Need AC, Shianna KV, et al. Long-Range LD Can Confound Genome Scans in Admixed Populations. The American Journal of Human Genetics 2008 Jul;83(1):132–135. https://doi.org/10.1016/j.ajhg.2008.06.005.
32. Danecek P, Auton A, Abecasis G, Albers CA, Banks E, DePristo MA, et al. The variant call format and VCFtools. Bioinformatics 2011 Jun;27(15):2156–2158. https://doi.org/10.1093/bioinformatics/btr330.
33. Kuhn RM, Haussler D, Kent WJ. The UCSC genome browser and associated tools. Briefings in Bioinformatics 2012 Aug;14(2):144–161. https://doi.org/10.1093/bib/bbs038.
34. Haeussler M, Zweig AS, Tyner C, Speir ML, Rosenbloom KR, Raney BJ, et al. The UCSC Genome Browser database: 2019 update. Nucleic Acids Research 2018 Nov;47(D1):D853–D858. https://doi.org/10.1093/nar/gky1095.
35. König IR, Loley C, Erdmann J, Ziegler A. How to Include Chromosome X in Your Genome-Wide Association Study. Genetic Epidemiology 2014 Jan;38(2):97–103. https://doi.org/10.1002/gepi.21782.
36. Continuum Analytics, Inc . Package, dependency and environment management for any language—Python, R, Ruby, Lua, Scala, Java, JavaScript, C/ C++, FORTRAN, and more.; 2017 (accessed October 16, 2020), https://docs.conda.io/en/latest/.
37. McLay R, Schulz KW, Barth WL, Minyard T. Best practices for the deployment and management of production HPC clusters. In: State of the Practice Reports on - SC '11 ACM Press; 2011. p. 1–11. https://doi.org/10.1145/2063348.2063360.
38. International HapMap Consortium, Frazer KA, Ballinger DG, Cox DR. A second generation human haplotype map of over 3.1 million SNPs. Nature 2007 Oct;449(7164):851–861. https://doi.org/10.1038/nature06258.
39. Kitts A, Sherry S. The Single Nucleotide Polymorphism Database (dbSNP) of Nucleotide Sequence Variation. In: McEntyre J, Ostell J, editors. The NCBI Handbook Bethesda (MD), US: National Center for Biotechnology Information; 2011.p. 1–34. Available from https://www.ncbi.nlm.nih.gov/books/NBK21088/.
40. Purcell S, Neale B, Todd-Brown K, Thomas L, Ferreira MAR, Bender D, et al. PLINK: A Tool Set for Whole-Genome Association and Population-Based Linkage Analyses. The American Journal of Human Genetics 2007 Sep;81(3):559–575. https://doi.org/10.1086/519795.
41. Li H, Handsaker B, Wysoker A, Fennell T, Ruan J, Homer N, et al. The Sequence Alignment/Map format and SAMtools. Bioinformatics 2009 Jun;25(16):2078–2079. https://doi.org/10.1093/bioinformatics/btp352.

Personal Cover
Click here to access/download;Personal Cover;Coverletter_revisions2.pdf

Kiel University
Christian-Albrechts-Universität zu Kiel
Faculty of Medicine

Institute of Clinical Molecular Biology , Rosalind-Franklin-Str. 12, 24105 Kiel

To
Dr. Scott Edmunds
Editor of *GigaScience*

ikmb
Institut für Klinische Molekularbiologie

Institute of Clinical Molecular Biology
Rosalind-Franklin-Str. 12
24105 Kiel

www.ikmb.uni-kiel.de

Board of Directors
Prof. Dr. Stefan Schreiber
Prof. Dr. Andre Franke
Prof. Dr. Philip Rosenstiel

Contact	E-mail
Prof. David Ellinghaus	d.ellinghaus@ikmb.uni-kiel.de
Dr.-Ing. Jan Christian Kässens	janchristian.kaessens@uksh.de
Dr.-Ing. Lars Wienbrandt	l.wienbrandt@ikmb.uni-kiel.de

Date
May 14, 2021

Revised manuscript; Submission ID GIGA-D-20-00360: "BIGwas- Single-command Quality Control and Association Testing for multi-cohort and biobank-scale GWAS/PheWAS data"

Dear Dr. Edmunds,

please find enclosed our revised manuscript and point-by-point response that addresses the reviewers' concerns. We hope that we have been able to address all of the concerns expressed.

We registered our software applications in the bio.tools and SciCrunch.org databases and obtained biotools and RRID (Research Resource Identification Initiative ID) identifiers. We have included these in our manuscript.

- bio.tools: https://bio.tools/bigwas_qc_pipeline and https://bio.tools/bigwas_assoc_pipeline
- The SciCrunch Research Resource Identification Initiative ID(RRID) are SCR_019240 and SCR_01924.

Yours faithfully,

Jan Christian Kässens, Ph.D. Lars Wienbrandt, Ph.D. David Ellinghaus, Ph.D.

e:Med SYSTEMS MEDICINE · SYS CID · Inflammation at Interfaces · RTG 1743 genes environment inflammation · META ORGANISMS · future ocean · human development in landscapes
