# Supplementary material for: BIGwas: Single-command quality control and association testing for multi-cohort and biobank-scale GWAS/PheWAS data
Source: Gigascience. 2021 Jun 29;10(6):giab047. doi: 10.1093/gigascience/giab047 (PMC8239664; doi:10.1093/gigascience/giab047)
Supplement: giab047_Response_to_Reviewer_Comments_Original_Submission [file giab047_response_to_reviewer_comments_original_submission.pdf]

Author's Response To Reviewer Comments

Close

Point-by-point reply for Submission ID GIGA-D-20-00360, “BIGwas - Single-command Quality Control and Association Testing for biobank-scale GWAS/PheWAS data”

Reviewer Comments:

Reviewer 1

In this paper, the authors present an automatic quality control pipeline for genotyped data using the nextflow language, making it easier to perform large scale genome wide association studies. This is a welcoming addition to the field, simplifying the usual tedious and oftentimes complicated quality control procedures.

We thank the Reviewer for the supportive feedback and interest in our implementation.

On our official GitHub tutorial websites https://github.com/ikmb/gwas-qc/ and https://github.com/ikmb/gwas-assoc, we have updated our software and documented the new software features in the Master Branch for the review process.

However, as of now, I was unable to run the proposed pipeline on the provided example dataset, and thus unable to provide an in-depth review on the performance / validity of the pipeline at the current stage.

We regret this and would like to know at which point in the instructions the problems occurred. For both the QC Module and Association Module, we have set up a dedicated Github webpage which, among other things, has a "Quick Start" guide at the beginning (https://github.com/ikmb/gwas-qc and https://github.com/ikmb/gwas-assoc) consisting of four short steps: (1) download the sample dataset (2) unpack the dataset, (3) QC Module execution with the example data set, which automatically installs the pipeline locally, (4) optional: execution of the Association Module using the example results of the QC Module and a direct link to the Assoc Module with more information about changing default parameters.

We would appreciate if reviewer #1 could run the “Quick Start" guide once and let us know if there may have been any technical issues with starting the pipeline.

Below are some of my comments based on the content of the paper and also my experience in try-ing to use the pipeline:

1. Under the quality control module, the authors suggested that all markers are matched with known variant database beforehand to bring the data to the same marker content. However, would-n't this remove all customized markers?

We agree with the reviewer that matching variants with known marker ID from external databases is an

important issue. In our implementation, variants of a custom chip are not lost during the matching step. The following sentence from the manuscript, section “Matching of Variant Identifiers with External Databases”, already describes how we handle custom-array variants without existing marker IDs in external databases: “If no match could be found in the database, the original variant name is retained and prefixed with “unk_” to indicate that neither name, position, alleles nor strand alignment could be verified, which is often the case if the content is custom chip content.”

2. Is it really necessary to perform the HWE test on all permutation of batch combinations? Would it be enough to perform HWE in each batch once, then the whole data set once? If we only performed once in each batch, then once in the whole dataset, then this shouldn't be too computa-tionally intensive.

We apologize if we have been unclear about this. The (admittedly short) answer is already given in the following sentence of the manuscript: “Batch-wise p-values are reported to find variants that fail HWE, among others, in a single control batch (but not in other control batches), in two or more con-trol batches and in the whole collection of controls.”

What we do is that we test for deviations from HWE for each individual SNP variant in three ways: (a) HWE in each control batch individually, (b) in the entire control data set, and (c) in the entire control data set without one of the control batches. Thus, the number of HWE tests increases line-arly with the number of control batches, and we are not performing a permutation of all control batches, which would indeed be very time-consuming. We then look for individual variants that pass the HWE P-value significance threshold in one of the following three ways: (i) the whole control da-taset shows deviation from HWE, (ii) two or more control batches each show deviation from HWE, (iii) the whole control dataset without any of the control batches shows deviation from HWE. In all three cases, the variant is removed from the dataset. However, if case (iii) shows that after remov-ing the control batch with the smallest (significant) HWE P-value, the total HWE P-value is no longer significant, then only the genotypes of the control batch with the smallest (significant) HWE P-value are set to "Missing". By doing so, we generally allow to keep variates that have been "badly geno-typed" in only a single control batch.

3. Based on the content of the paper, it seems like this pipeline is specifically designed for case control phenotypes in European samples? Is it possible to extend the pipeline to continuous trait and also for non-European samples?

With the help of our colleague Dr. Lars Wienbrandt we have extended the quality control module for an analysis of quantitative traits. Therefore, Lars Wienbrandt is now also co-author on the manuscript. To clarify this, we now write in the Abstract: “Here we present BIGwas, a portable, fully automated QC and association testing pipeline for large-scale binary and quantitative trait GWAS data provided by biobank resources”.

Now the entire pipeline (QC module and association module) works for binary as well as quantitative traits. For a QC based on quantitative traits, in the PLINK input Fam file, only the 6th column must be specified as a quantitative trait (for more information, see also http://zzz.bwh.harvard.edu/plink/data.shtml#ped). We added this information to our tutorial website https://github.com/ikmb/gwas-assoc. This means that, as implemented in the PLINK program, it is assumed to be a quantitative trait as soon as values different from 1 (control), 2 (case) or 0 / -9 (missing) are defined in the 6th column of the PLINK Fam file. In this case, the HWE test is performed on all individuals (instead of only on the controls of a case-control study). In the Assoc module only the parameter --trait has to be set to "quantitative" instead of “binary” (see https://github.com/ikmb/gwas-assoc, section “Parameters”). PLINK as well as SAIGE will then automatically perform an association analysis for a quantitative trait, that means, in the association analysis a linear regression and a linear mixed model association analysis are performed instead of a logistic regression and a logistic mixed model association analysis. The information above is briefly described in sections “Quality Control Module” and “Association Testing Module”.

In principle, the QC module can be used for any European and non-European population. However, for the special case of an intended trans-ancestry GWAS study (for example to analyze GWAS samples of European, Asian and African descent), we recommend that a separate quality control be performed for each ethnic group. We added this information in the manuscript at the end of section “Quality Control Module”.
The "ethnicity_predicted" column in the sample annotation file (see tutorial on https://github.com/ikmb/gwas-qc) is there, first, to help the user remember which ethnicity to assign the study participants to. Then, based on the numerically most frequently specified ethnicity in col-umn "ethnicity_predicted" the HWE test is performed only for controls of that ethnicity (or for all samples with that ethnicity if it is a quantitative trait). To better emphasize this, we added the follow-ing sentence to our QC tutorial website: “Based on the numerically most frequently specified ethnici-ty in column "ethnicity_predicted" the HWE test is performed only for controls of that ethnicity (or for all samples with that ethnicity if it is a quantitative trait).”

We further added the following sentence to section “Test for Hardy-Weinberg-Equilibrium” in the manuscript: “Hardy-Weinberg equilibrium tests are performed for controls only in a case-control study (and for all individuals if a quantitative trait is to be considered)."
Afterwards, based on the total GWAS dataset, the PCA analysis with the 1000 Genomes reference samples removes study samples (per default) which have values smaller than the median plus/minus five times the interquartile range (median ±5×IQR; parameter adjustable) of all GWAS study samples for the first two PCs. To better emphasize this, we adjusted the following sentence in section “Controlling for population stratification and other confounders” in the following way: “Individ-ual GWAS samples (grey crosses) with values smaller than the median plus/minus five times the interquartile range (median ±5×IQR; samples outside the red square in PCA plot) represent PCA outliers for the first two PCs and are automatically removed (per default; adjustable)”. For example, if a sufficiently large number of European and non-European origin GWAS samples are in the da-taset, then this approach will result in no outliers being detected. However, if there are only a small number of non-European GWAS samples in a large dataset of European GWAS samples (or vice versa), this will have the desired effect of detecting the non-European GWAS samples as PCA out-liers in the dataset of European GWAS samples (or vice versa). We recommend that the user con-siders the sample selection regarding the ethnicity in advance of the GWAS study, because even the best automated QC pipeline cannot replace a coherent study design.

Also, the Association Module is not specialized for individuals with European ancestry and can be used for cohorts of any ancestry as long as they represent a reasonably homogeneous study pop-ulation. However, for the special case of a intended trans-ancestry association analysis, we rec-ommend either performing a single association run for each ethnic group or using specific trans-ancestry association testing programs, for example, MANTRA (PubmedID 22125221) or modified random effect model (RE2C; PubmedID 28881976), that account for differences in allelic effects resulting from differences in linkage disequilibrium (LD) between (the user-defined) populations. To make this clear, we’ve already written in the Discussion section that “BIGwas is not yet suitable for GWAS analysis of family data or GWAS analysis across populations of worldwide ethnicities.”

4. Will pruning be performed before performing the heterozygousity check?

The distribution of mean heterozygosity (excluding sex chromosomes) across all individuals is cal-culated based on all quality-controlled genetic markers that passed “SNP QC I” and does not in-clude an additional pruning step.

5. Will the same 14,484 SNPs always be used for IBS and IBD estimation? If not, then it might be better to describe the pruning procedure as the 14,484 number is likely specific to the dataset used by the author.

In another test run for the so-called Immunochip (not mentioned in the manuscript) pruning ended up with 14,484 SNPs as the best suitable set of independent SNPs for PCA and the "Dupli-cate/Relatedness Detection" analysis. We apologize for this misleading statement in the manuscript and removed this number. Instead, the "Duplicate/Relatedness Detection" analysis uses the same set of variants that is dynamically determined for the PCA analysis as described in this sentence: "For all PCA, a set of independent (MAF >0.05) SNPs is calculated, excluding non-autosomes, vari-ants in LD (leaving no pairs with r2>0.2, within 50 kb windows) or within the extended major histo-compatibility complex (xMHC; chr6:25-34Mb), and 11 high-LD regions as described by Price et. al. [31]." We have revised the following text passage about IBS/IBD estimation as follows: "For robust duplicate/relatedness testing (identity-by-state (IBS) and identity-by-descent (IBD) estimation), we use the same set of independent variants, which was dynamically determined for PCA analysis (see above)."

6. Will the pipeline filter out samples with mismatch self-reported and genetically indicated sex information? And will the pipeline remove samples with sex aneuploidy?

No, the QC module will not filter out samples where there may be a discrepancy between self-reported and genetically determined biological sex. The reason for this is that common sex-detection methods, such as the method used by PLINK (http://zzz.bwh.harvard.edu/plink/summary.shtml#sexcheck), only use chromosome X variants and therefore, in our experience, do not work well in many individuals.

We rather recommend a verification between self-reported and genetically determined biological sex by means of normalized signal intensities of SNPs on chromosome X as well as chromosome Y simultaneously. However, because this step requires e.g. Illumina iDat files raw data with normal-ized signal intensities instead of genotypes in PLINK format, we have omitted this step in the QC Module. To clarify this, we have included the following sentence in the Methods section: "The QC module does not perform a check between self-reported and genetically determined biological sex based on chromosome X PLINK genotypes; we recommend using normalized signal intensities of SNPs on chromosome X as well as Y for this purpose."

The PLINK genotype and vcf files from SNP arrays and/or genotype imputation processes do not provide information about an abnormal number of sex chromosomes. For this reason, individuals with sex chromosome aneuploidies cannot be detected.

7. It is possible to convert BGEN data to VCF using PLINK2, which also natively support BGEN.

It is true that the UK Biobank's latest GWAS data release contains, in addition to unimputed geno-type data in PLINK format, imputed data in the BGEN format instead of the much more common VCF format. However, since the more common VCF format is a generic format used by biobanks and genotype imputation programs (TOPMed and Sanger Imputation server output, among others) worldwide, and because the conversion from BGEN to VCF took an extremely long time and cost an extremely large amount of hard disk space with UK Biobank's cohort size for our benchmark purposes, we chose to drop UK Biobank's BGEN support by default. Thus, in addition to SAIGE, we can continue to use PLINK as the de facto gold standard tool for regression-based association analyses in our Association Module, even for the analysis of UK Biobank, because PLINK cannot directly read BGEN for association analyses. Because UK Biobank is, to our knowledge, the only dataset where the BGEN format is used by default, we offer a simplified the association pipeline for the UB Biobank case and a set of scripts to perform SAIGE association testing directly on UKB BGEN files on https://github.com/ikmb/gwas-assoc", see section "UK Biobank Proof-of-concept".

8. With relatedness filtering, can the pipeline automatically calculate the full relatedness matrix? E.g. for UK Biobank, the full 500,000 x 500,000 matrix? Or does it perform filtering within each 2,000-sample cluster, then merge the post-filtering sample and repeat until no-relatedness remain? In the latter case,

how do you ensure the maximum number of samples are retained? Is `--rel-cutoff` used?

Indeed, our QC Module calculates the full relatedness matrix from all 488k UK Biobank samples (or even from 976k samples, for benchmark results see Table 2) without using Plink's `--rel-cutoff` (i.e. there is no exclusion of one member of each pair of samples with observed genomic relatedness greater than a given cutoff value). Instead of using n-sample clusters for parallelization, we used PLINK's IBD interface using `--genome` for calculating the full relatedness matrix and run all combi-nations of sample pairs in parallel chunks using different compute jobs. The number of jobs running in parallel is dynamically selected based on the number of samples (see also Figure 2).

9. It is rather surprising that the whole pipeline requires 7 days to complete. Which is the most time-consuming step? It'd be interesting to know. I'd imagine the PCA and Relatedness matrix re-quire the most time to compute.

We are of the opinion that a complete QC and association analysis in only 7 days is unusually fast for a GWAS dataset of size 974,818 samples and 92 million variants with a limited use of 150 CPU cores for computation. This is actually possible because we dynamically parallelized as many steps of QC and association analysis as possible with respect to the number of samples (n) and variants (m; see also Figure 2 and 3).

In terms of CPU time, most CPU hours are consumed during our extensive Hardy-Weinberg analy-sis in the part where we re-calculate the HWE for each leave-one-batch-out permutation. Although this part is highly parallelized, it still accounts for a large fraction of the total time because we limit the number of active parallel jobs on our high-performance cluster (HPC) to 150 (by default; but configurable). Another time-consuming step is merging the input datasets with reference genotype data sets in preparation for PCA, i.e., merging the input dataset with the HapMap and 1000 Ge-nomes files, which is not parallelizable by default with Plink 1.9 and is not currently available in Plink 2. Interestingly, the PCA and relatedness matrix computation consumes less than 20% of the wall clock time. One reason for this is the fact that we use the FlashPCA2 program for PCA (Abraham et al., 2017, PubMedID 28475694), which can perform PCA on 1 million individuals faster than other competing approaches, while requiring substantially less memory (see also section "Sample QC" in our manuscript).

Below are some comments regarding the implementation of the pipeline:
1. As of now, running the pipeline require extensive knowledge of the nextflow language and behavior. For example, any cluster options written in the local *.config files are going to be overwrit-ten by the $HOME/.nextflow/assets/ikmb/gwas-qc/nextflow.config file (which has process.executor predefined as slurm).

We thank the Reviewer for pointing out this issue with the process executor variable of the Slurm batch system. Users do not need much experience with the Nextflow programming environment to start our pipeline and this default setting of the Slurm batch system was not known to us until now. We have now removed the setting for this process executor variable in our current source code so that the pipeline runs locally by default. The process for setting the correct executor for the HPC system is now explained in the readme file, see section "UKSH medcluster configuration". This change must be done in the nextflow config file which is located at $HOME/.nextflow/config.

2. On some HPC, the $HOME directory can have limited space (e.g. one from my institute only have 20GB). Given that the current pipeline use $HOME for temporary output, users can quickly run out of spaces. To avoid that, users will need to do export _JAVA_OPTIONS=-Djava.io.tmpdir=

We assume that this is a misunderstanding because no change needs to be made to the Java in-stallation. In Nextflow it is quite easy to set the NXF_WORK environment variable to a location of your choice (https://www.nextflow.io/docs/latest/config.html). Variable NXF_WORK points to a user defined directory where working files are stored (usually your scratch directory). We thank the Re-viewer for this advice and have updated our readme online documentation to explain where to set the Nextflow working folder.

3. When I tried to run the pipeline, I cannot get pass the RS.nf stage, which gives me an er-ror: FATAL: Cached File Hash(sha256.33fc75ba032f40e5df1292914c250482a4fbe34d274bea995bcaba0b82c3befc) and Expected Hash(sha256.707eb6df0d5b7b93ea01f6bfa34fffc4a7efb62541d8194b815d9359d9510091) does not match

We regret the occurrence of this error message and we were also wondering how such an error message can occur. We have investigated different Nextflow versions and user forums and it seems like that there is a problem in Nextflow that is (very rarely) triggered when the directory in $NXF_WORK or the Java tempdir with the above_JAVA_OPTIONS is set to a Samba/CIFS net-work share or sometimes to a poorly configured service that introduce file caching incoherence (deprecated NFS, misconfigured Lustre mounts, S3 mounts). We have now included a few worka-round solutions on how to set up $NXF_WORK on a SAMBA shared directory in our readme online documentation, see section “Cache issues”. Nevertheless, we do not recommend working on a SAMBA directory when specifying $NXF_WORK.

4. One disadvantage of using nextflow is its inability of deleting intermediate files. Deleting the intermediate files will invalidate -resume, requiring users to restart the whole pipeline from scratch instead of picking up from where they end. Within the current pipeline, there are around 50+ in-stance of `--make-bed`, which generates large bed files. Instead of using `--make-bed`, it might be better to use `--make-just-fam` and `--write-snplist` in place of `--make-bed` whenever possible such that only the ID of SNPs and samples were reported between steps instead of writing out the whole genotype matrix. `--extract`, `--exclude`, `--keep` and `--remove` can then be used to per-form cheap on the fly filtering on subsequent steps. This should also significantly reduce IO burden and thus improve performance. However, it is acknowledged that `--make-bed` is still required for operations such as merging the different batch.

We thank the reviewer for these good suggestions for changes. Now we have switched to these lightweight options where possible, resulting in a 20% reduction in intermediate file size for our ex-ample dataset. We are very happy about that. However, there are still a few instances of --make-bed where the intermediate genotype matrix is used as input for non-PLINK tasks without the ability to use exclude/keep lists. Further, some time-consuming steps such as merging PLINK flles before PCA remain and unfortunately cannot be bypassed. Interestingly, in terms of runtime, we have not yet been able to detect any measurable runtime reductions as a result of the changes, which may also be due to our fast file storage system.

5. Given the design of the current pipeline, it might be beneficial to use the latest DSL 2.0 fea-ture of nextflow, which allow piping and modulating different processes. This should substantially improve the organization of the scripts, avoid the need to calling another nextflow instance within the current nextflow pipeline.

We agree that the latest DSL 2.0 would potentially be beneficial to the overall structure of the pipe-line to make future processes easier to modify. However, this pipeline is the result of a lot of imple-

mentations we have done over the last few years and already existed at its core when DSL 2.0 was first introduced as a first stable release in June 2020. A completely new pipeline would certainly use DSL 2.0, but the DSL version is not critical for the user. Re-implementing the pipeline in DSL 2.0 with the typical several rounds for debugging would require an enormous amount of time without any apparent benefit we see for the user.

6. It might be beneficial to provide a command line interface (e.g. utilize the $param object from nextflow) so that users can easily specify parameters with `--output`, `--datasets` etc.

Since our pipeline supports multiple datasets, where each dataset can contain multiple batches, we make a lot of use of arrays in our configuration files. Unfortunately, Nextflow does not support spec-ifying array contents on the command-line. However, it is possible to specify simple (i.e., non-array) parameters on the command-line that would otherwise appear in the pipeline.config file, in particular the `--output` parameter and the `--qc_config` parameter.

Reviewer 2

Drs. Kässens and Ellinghaus present BIGwas, a single-command analysis tool to jointly run data quality control and association analysis in the context of genome-wide association studies (GWAS). The Authors should be commended for dedicating time and releasing such a software, as there is great need of standardized quality control procedures in the GWAS field.

We thank the Reviewer for the supportive feedback and interest in our software.

On our official GitHub tutorial websites https://github.com/ikmb/gwas-qc/ and https://github.com/ikmb/gwas-assoc, we have updated our software and documented the new software features in the Master Branch for the review process.

I would like to point the authors' attention towards some points that, in my opinion, deserve some clarification.

- When I started reading the manuscript it took me a while before I realised that this is a tool for quality control from the individual data of each participant. Perhaps I was misled by the fact that many GWAS quality control softwares only focus on post-GWAS results. Perhaps this deserves immediate clarification at the beginning of the paper.

We thank the reviewer for this valuable comment. To further clarify this point, we have included the following underlined text at the end of the introduction: "… there are still no easily executable and reproducible software pipelines available for performing large-scale quality control (QC) and asso-ciation tests on a large number of individual (large-scale) GWAS or PheWAS data sets totaling mil-lions of GWAS samples.

- Linked to the previous discussion, I would ask the authors to put the use of BIGwas a bit better into the context of consortia that include hundreds of studies, some very large like UKBB, others very small (1000-2000 samples): is there a real advantage to using BIGwas, given that many stud-ies have their pipelines already implemented and many are not willing to change them? What might be the advantages/disadvantages?

We agree with the reviewer that BIGwas best realized to its full potential in the context of a mega-GWAS study, where the user has to perform quality control (QC) analysis across dozens of co-horts simultaneously. To better emphasize this, we have included the following underlined sentence at the end of the introduction: “In particular, parallel and consistent processing and analysis of doz-ens of individual GWAS cohorts in the context of a mega-GWAS analysis is enormously time-consuming and can quickly become unmanageable if processed manually.” In addition, we have modified the title to read: "BIGwas- Single-command Quality Control and Association Testing for multi-cohort and biobank-scale GWAS/PheWAS data.”

On the other hand, BIGwas also demonstrates its full potential in the execution of single-cohort GWAS studies: Due to the Singularity container and Nextflow pipeline technology, extensive installa-tion is not required and only a single command is needed to perform a complete QC and/or associa-tion analysis. This makes BIGwas thus also interesting for users who may only want to perform a single-cohort GWAS study only once as part of a larger multi-omics study. This saves these users lots of training time to acquire knowledge in the field of genome-wide association studies (GWAS).

- Since BIGwas is implemented in the context of GWAS consortia, in my opinion it would be appro-priate not only to compare it with H3Agwas but also with mixed approaches (e.g. study-specific pipelines for post-GWAS analysis and quality control, eg GWAtoolbox or similar). If a formal com-parison is not feasible, perhaps one could at least discuss advantages/disadvantages of alternative approaches.

Due to the above-mentioned reasons, we believe that BIGwas can show its potential in both meg-aGWAs and single-cohort GWAS analyses. We also understand well that there is a desire for a comprehensive comparison of BIGwas with existing GWAS analysis packages in R that can be used to manually perform QC or GWAS association analysis steps. However, we would then turn our comparison to existing software programs and packages, with which one can execute certain subtasks of the analysis pipeline only by a manual call of certain functions. The intention of our pipe-line development was to allow QC and Assoc testing to be performed in a standardized and repro-ducible manner with a single command, and for users new to GWAS to not have to spend a lot of time dealing with a variety of software packages when the goal is to easily perform a state-of-the-art GWAS analysis.

We agree that BIGwas may not be of as much interest to the very experienced GWAS user who wants full control over all possible packages and analysis steps and wants to build her/his own pipeline. Therefore, in order to highlight the advantages and disadvantages of using BIGwas in comparison to existing R packages we have added the following sentence to the Discussion sec-tion: “BIGwas differs from existing GWAS data processing packages in R in that it allows standard-ized, reproducible, and high-throughput processing of GWAS datasets via a single command, whereas existing GWAS R packages allow for more flexible processing, for example, with respect to the order of processing steps.”

- It seems that a limitation of BIGwas is the very strong reference to the IBDGC and Severe Covid-19 GWAS group consortia's protocols. This affects many aspects of the proposed analysis plan. For example, it is very common to find groups that do not use PLINK at all, neither at the quality control level nor at the analysis level; how should such groups behave in the management of e.g. the X chromosome or other steps if they wanted to use BIGwas?

Now we have removed the reference to the IIBDGC Consortium and the GWAS Group in the ab-stract, as the QC and association testing protocol applied is not only applicable to the analysis of diseases from these alliances but can be applied to various binary and quantitative traits in general. We agree that mentioning these disease-specific alliances in the Abstract may confuse the reader.

We cannot rule out the possibility that there are very experienced GWAS analysts who prefer R-only packages, already have own their own software pipeline, and want to avoid PLINK entirely. On the other hand, we see no genuine reason not to use PLINK for specific subtasks such as IBD/IBD estimation, as it

is the most widely used and robust C++ software tool available for GWAS studies, with an excellent code base and extensive use of bit-level parallelism (Chang et al., Gigascience. 2015 Feb 25;4:7). We still think we can convince even the very experienced GWAS analysts to use BIGwas because, as noted, we offer a complete QC and association analysis including association analysis for the X chromosomal regions via a single command. If the QC module of BIGwas is not of interest, the very experienced GWAS analyst might still be interested in the Association Module of BIGwas. The User could then easily use only the Association Module of BIGwas, since the two modules of BIGwas run completely independently of each other.

- Apparently, the pipeline is thought for binary trait analysis (cases and controls). Is it also suitable for quantitative traits? Can the Authors specify this better?

With the help of our colleague Dr. Lars Wienbrandt we have extended the quality control module for an analysis of quantitative traits. Therefore, Lars Wienbrandt is now also co-author on the manuscript. To clarify this, we now write in the Abstract: “Here we present BIGwas, a portable, fully automated QC and association testing pipeline for large-scale binary and quantitative trait GWAS data provided by biobank resources”.

Now the entire pipeline (QC module and association module) works for binary as well as quantitative traits. For a QC based on quantitative traits, in the PLINK input Fam file, only the 6th column must be specified as a quantitative trait (for more information, see also http://zzz.bwh.harvard.edu/plink/data.shtml#ped). We added this information to our tutorial website https://github.com/ikmb/gwas-assoc. This means that, as implemented in the PLINK program, it is assumed to be a quantitative trait as soon as values different from 1 (control), 2 (case) or 0 / -9 (missing) are defined in the 6th column of the PLINK Fam file. In this case, the HWE test is performed on all individuals (instead of only on the controls of a case-control study). In the Assoc module only the parameter --trait has to be set to "quantitative" instead of “binary” (see https://github.com/ikmb/gwas-assoc, section “Parameters”). PLINK as well as SAIGE will then automatically perform an association analysis for a quantitative trait, that means, in the association analysis a linear regression and a linear mixed model association analysis are performed instead of a logistic regression and a logistic mixed model association analysis. The information above is briefly described in sections “” and “Association Testing Module”.

- It is unclear whether the IBD QC step will exclude related individuals or if related individuals can be retained in the analysis by using linear mixed modeling approaches. These models could also incorporate a random batch effect, for instance, thus making the batch effect QC step unnecessary.

We thank the reviewer for this valuable advice. Per default we determine the relatives using IBD/IBS analysis, remove them from the final dataset, and output the list of relatives in an additional text file during SampleQC so that the user could add them back later if desired. For the QC Module, we have now implemented an additional switch "--keep_related" which, if set, keeps the relatives in the final output files from QC. This parameter can be specified in the dataset-specific configuration, the general pipeline configuration, or directly on the command line. For the Association Module, the user can still decide by specifying the samples in the fam file, which samples she/he wants to use for the association analysis.

- In addition, to PLINK and SAIGE, there are other popular GWAS software such as BOLT-LMM, REGENIE, and others. Can they be implemented within the BIGwas analysis pipeline?

In principle, yes. We decided not to add BOLT-LMM (purely linear mixed model without providing a logistic mixed model for binary traits) in addition to PLINK logistic/linear regression and SAIGE lo-

gistic/linear mixed model analysis in our Association Module because, on the one hand, it further increases the overall runtime as well as the number of jobs running in parallel, and, on the other hand, we don't expect too much gain from an additional linear mixed model tool such as BOLT-LMM, as the developers of BOLT-LMM themselves state the following on their website: “We rec-ommend BOLT-LMM for analyses of human genetic data sets containing more than 5,000 samples. The algorithms used in BOLT-LMM rely on approximations that hold only at large sample sizes and have been tested only in human data sets. For analyses of fewer than 5,000 samples, we recom-mend the GCTA or GEMMA software. We also note that BOLT-LMM association test statistics are valid for quantitative traits and for (reasonably) balanced case-control traits. For unbalanced case-control traits, we recommend the SAIGE software.”

REGENIE is an interesting new tool recently published on bioRxiv. REGENIE allows parallel associ-ation analysis of multiple phenotypes simultaneously from a single input dataset such as UK Bi-obank, which is particularly interesting when each association analysis always refers to the same GWAS input dataset and only the phenotype information changes per run. This type of analysis requires of a range of phenotypes for individuals in the same dataset, as is the case with UK Bi-obank. Therefore, we see REGENIE as a special tool that we could add in the future as an addition-al association Nextflow module for multi-phenotype analysis based on a single GWAS input dataset.

- GWAS and PheWAS are discussed under the same umbrella; however, the whole pipeline is pre-sented and discussed only with regard to GWAS; please outline key differences between the two approaches or limit to GWAS

We agree with the reviewer that the focus of the benchmark here was to demonstrate the simplicity and reproducibility of a multi-cohort mega-GWAS QC and large-scale association analysis. Now it is not a far step to create a PheWAS resource (e.g. as provided by the Michigan Genomics Initiative (MGI), see http://pheweb.sph.umich.edu/) based on the genetic data when numerous different phe-notype information are available for the same individuals. For example, to perform a (genome-wide) PheWAS analysis with five different traits for one and the same data set, it is sufficient to create five different PLINK fam files for binary or quantitative traits for the same GWAS input data set and to call the pipeline five times each by a single command.

To better highlight this PheWAS usability, which now offers itself following the GWAS analyses with BIGwas, we have included the following sentence in the Introduction section about existing PheWAS resources: “To create a (genome-wide) PheWAS resource of this type, BIGwas can be used alongside the QC and mega GWAS analysis capability to test for association with a wide variety of traits in parallel or sequentially (requiring only one single program call per trait on a HPC) when nu-merous different phenotype information is available for the same individuals.”

- Please, explain all jargon. For instance, most audience might be unfamiliar with terms such as "containerized".

We apologize for assuming the term "containerized" to be familiar to those reading the manuscript. We have revised the Abstract and the first sentence of the Discussion as follows: “Here we present BIGwas, a portable, fully automated QC and association testing pipeline for large-scale GWAS data provided by biobank resources.” and “We have introduced BIGwas, a portable (i.e. Singularity con-tainer-based), fully automated GWAS quality control (QC) and association pipelines in Nextflow that can be executed with a single command without manual installation by the user.

- In the Abstract, I would suggest to keep the approach general, avoiding referring to specific situa-tions that might soon become outdated (eg: "the number of GWAS samples is twice as large as the current GWAS data release of the UK Biobank".

We thank the reviewer for this advice and have revised this sentence in the abstract as follows:” For a GWAS analysis with 974,818 individuals and 92 million genetic markers, BIGwas takes around 16 days on a small high performance compute system (HPC) with 7 compute nodes to per-form a complete GWAS QC and association analysis protocol.”

- Please, review text for typos. For instance, I found "per centage" in place of "percentage".

We have corrected this and other typos.

Close
