# Supplementary material for: BIGwas: Single-command quality control and association testing for multi-cohort and biobank-scale GWAS/PheWAS data
Source: Gigascience. 2021 Jun 29;10(6):giab047. doi: 10.1093/gigascience/giab047 (PMC8239664; doi:10.1093/gigascience/giab047)
Supplement: giab047_Response_to_Reviewer_Comments_Revision_1 [file giab047_response_to_reviewer_comments_revision_1.pdf]

Author's Response To Reviewer Comments

Close

Point-by-point reply for Submission ID GIGA-D-20-00360, “BIGwas- Single-command Quality Control and Association Testing for multi-cohort and biobank-scale GWAS/PheWAS data”

Reviewer Comments:

Reviewer #1:

In this update, the authors have addressed most of my concerns. In addition, the update to the README on their github sufficiently extensive, and I can now run the example data set after some minor changes. The paper should now ready to be published.

We thank the reviewer for the supportive feedback and the suggestions for modifications.

Some minor comment regarding the paper and the script:
1. Under "Assignment of variants to Pseudo-autosomal Regions", correct styling of "Plink" should be "PLINK"

We now write "PLINK" instead of "Plink" in all places in the manuscript.

2. The `qc-assoc-assets.sif` container is unsigned. As a result, singularity pull will fail and re-quire user to pull the singularity image themselves. Users will also need to set `singularity.cacheDir` variable in the ` ~/.nextflow/assets/ikmb/gwas-qc/nextflow.config` file. Not sure why setting this var-iable in the local nextflow.config doesn't work.

We now have created a signed version of the sif container image:
https://cloud.sylabs.io/library/_container/5f43b624b1793638c11379be

In principle, Singularity also loads unsigned container files (default setting in Singularity). Generally it is not necessary to set the variable "singularity.cacheDir" in Nextflow, see https://www.nextflow.io/docs/latest/config.html#scope-singularity. However, we recommend to set the singularity cache directory in the configuration file $HOME/.nextflow/config, see also paragraph "Shared Singularity Cache" in our readme file at https://github.com/ikmb/gwas-qc/, to avoid having to download the image files over and over again. Please note that the cache directory must be accessible from all compute nodes when running BIGwas on a high-performance compute (HPC) cluster, see also paragraph "Shared Singularity Cache" in our readme file.

3. label 'phase' for multiple processes were not defined, causing pipeline to fail when using with HPC scheduler. It seems like `phase` should use at least 8 CPUs (required by flash-pca2_pruned_1kG process), but the default is 1.

We thank the reviewer for this valuable comment. The process you mentioned (flash-pca2_pruned_1kG) has indeed defined its own label to account for the CPU usage of flashpca2. However, the 'phase' label is reserved for the start of the phases (Rs, SNPQCI, SampleQC, etc.) and probably does not help with your pipeline crash. After reviewing the relevant source code, we have concluded that there may be either a configuration issue on your end or a problem with the pipeline control mechanism we are using. We would be happy to help you here and would appreciate it if you could post this issue in our public issue tracker at https://github.com/ikmb/gwas-qc/issues. It would also be very helpful if you could attach there 1) the ".nextflow.log" file generated by your crashed run, 2) the exact error message, and 3) the ".command.log" file located in the "working directory" that Nextflow shows in the error report, as well as 4) some details about your installation, for example, which job scheduler you are using, and how you have set up your personal Nextflow configuration.

Some suggestion that should:
- Considering that the HWE took up the most computation time, then a possibly quicker alter-native might be the following:
1. Run `--hardy` once in each cohort independently (or maybe --frqx for quantitative traits).
2. Extract the GENO column from the .hwe file to obtain the A1 hom, het and A2 hom count for the control samples
3. For each SNP, you should now be able to calculate the number of A1 hom, het and A2 hom count of control samples with all possible cohort combinations.
4. Use the standalone hwe p-value calculation software here: https://github.com/chrchang/stats to calculate the p-value for each SNP and cohort combina-tion
- The benefit of this approach is that the calculation of the A1, A2 and het count for each co-hort combination is now reduced to a single summation and the p-value should be able to be calcu-lated rather quickly. Hopefully this can help to speed up the current software

We thank the reviewer for this suggestion, which we will be happy to examine in more detail in the future. Such a major change to the code requires again extensive testing of the HWE subroutine for binary and quantitative traits, which is currently running without errors. Furthermore, we are currently using our implementation in R to additionally perform the HWE calculation and on-the-fly filtering for PLINK files with respect to the specified "ethnicity" from the annotation file, which has proven to be very effective to implement in R without having to further preprocess the PLINK files. Replacing the subroutine with a different source code also requires adapting the interface for generating the DeFinetti and HWE output figures. For this reason, we would like to leave the current implementation as it is.

Close
