# Supplementary material for: BIGwas: Single-command quality control and association testing for multi-cohort and biobank-scale GWAS/PheWAS data
Source: Gigascience. 2021 Jun 29;10(6):giab047. doi: 10.1093/gigascience/giab047 (PMC8239664; doi:10.1093/gigascience/giab047)
Supplement: giab047_Response_to_Reviewer_Comments_Revision_2 [file giab047_response_to_reviewer_comments_revision_2.pdf]

Author's Response To Reviewer Comments

Close

We thank you for your comments and have adjusted the manuscript accordingly.

Close
