# Supplementary material for: BIGwas: Single-command quality control and association testing for multi-cohort and biobank-scale GWAS/PheWAS data
Source: Gigascience. 2021 Jun 29;10(6):giab047. doi: 10.1093/gigascience/giab047 (PMC8239664; doi:10.1093/gigascience/giab047)
Supplement: giab047_Reviewer_1_Report_Original_Submission — Shing Wan Choi -- 12/23/2020 Reviewed [file giab047_reviewer_1_report_original_submission.pdf]

Reviewer Report

Title: BIGwas - Single-command Quality Control and Association Testing for multi-cohort and biobank-scale GWAS/PheWAS data

Version: Original Submission **Date:** 12/23/2020

Reviewer name: Shing Wan Choi

Reviewer Comments to Author:

In this paper, the authors present an automatic quality control pipeline for genotyped data using the nextflow language, making it easier to perform large scale genome wide association studies. This is a welcoming addition to the field, simplifying the usual tedious and oftentimes complicated quality control procedures. However, as of now, I was unable to run the proposed pipeline on the provided example dataset, and thus unable to provide an in-depth review on the performance / validity of the pipeline at the current stage. Below are some of my comments based on the content of the paper and also my experience in trying to use the pipeline:

1. Under the quality control module, the authors suggested that all markers are matched with known variant database beforehand to bring the data to the same marker content. However, wouldn't this remove all customized markers?
2. Is it really necessary to perform the HWE test on all permutation of batch combinations? Would it be enough to perform HWE in each batch once, then the whole data set once? If we only performed once in each batch, then once in the whole dataset, then this shouldn't be too computationally intensive.
3. Based on the content of the paper, it seems like this pipeline is specifically designed for case control phenotypes in European samples? Is it possible to extend the pipeline to continuous trait and also for non-European samples?
4. Will pruning be performed before performing the heterozygousity check?
5. Will the same 14,484 SNPs always be used for IBS and IBD estimation? If not, then it might be better to describe the pruning procedure as the 14,484 number is likely specific to the dataset used by the author.
6. Will the pipeline filter out samples with mismatch self-reported and genetically indicated sex information? And will the pipeline remove samples with sex aneuploidy?
7. It is possible to convert BGEN data to VCF using PLINK2, which also natively support BGEN.
8. With relatedness filtering, can the pipeline automatically calculate the full relatedness matrix? E.g. for UK Biobank, the full 500,000 x 500,000 matrix? Or does it perform filtering within each 2,000-sample cluster, then merge the post-filtering sample and repeat until no-relatedness remain? In the latter case, how do you ensure the maximum number of samples are retained? Is `--rel-cutoff` used?
9. It is rather surprising that the whole pipeline requires 7 days to complete. Which is the most time-consuming step? It'd be interesting to know. I'd imagine the PCA and Relatedness matrix require the most time to compute.

Below are some comments regarding the implementation of the pipeline:

1. As of now, running the pipeline require extensive knowledge of the nextflow language and behavior. For example, any cluster options written in the local *.config files are going to be overwritten by the $HOME/.nextflow/assets/ikmb/gwas-qc/nextflow.config file (which has process.executor predefined as slurm).
2. On some HPC, the $HOME directory can have limited space (e.g. one from my institute only have 20GB). Given that the current pipeline use $HOME for temporary output, users can quickly run out of spaces. To avoid that, users will need to do export _JAVA_OPTIONS=-Djava.io.tmpdir=
3. When I tried to run the pipeline, I cannot get pass the RS.nf stage, which gives me an error: FATAL: Cached File Hash(sha256.33fc75ba032f40e5df1292914c250482a4fbe34d274bea995bcaba0b82c3befc) and Expected Hash(sha256.707eb6df0d5b7b93ea01f6bfa34fffc4a7efb62541d8194b815d9359d9510091) does not match
4. One disadvantage of using nextflow is its inability of deleting intermediate files. Deleting the intermediate files will invalidate -resume, requiring users to restart the whole pipeline from scratch instead of picking up from where they end. Within the current pipeline, there are around 50+ instance of `--make-bed`, which generates large bed files. Instead of using `--make-bed`, it might be better to use `--make-just-fam` and `--write-snplist` in place of `--make-bed` whenever possible such that only the ID of SNPs and samples were reported between steps instead of writing out the whole genotype matrix. `--extract`, `--exclude`, `--keep` and `--remove` can then be used to perform cheap on the fly filtering on subsequent steps. This should also significantly reduce IO burden and thus improve performance. However, it is acknowledged that `--make-bed` is still required for operations such as merging the different batch.
5. Given the design of the current pipeline, it might be beneficial to use the latest DSL 2.0 feature of nextflow, which allow piping and modulating different processes. This should substantially improve the organization of the scripts, avoid the need to calling another nextflow instance within the current nextflow pipeline.
6. It might be beneficial to provide a command line interface (e.g. utilize the $param object from nextflow) so that users can easily specify parameters with `--output`, `--datasets` etc.

Level of Interest

Please indicate how interesting you found the manuscript: Choose an item.

Quality of Written English

Please indicate the quality of language in the manuscript: Choose an item.

Declaration of Competing Interests

Please complete a declaration of competing interests, considering the following questions:

- Have you in the past five years received reimbursements, fees, funding, or salary from an organisation that may in any way gain or lose financially from the publication of this manuscript, either now or in the future?
- Do you hold any stocks or shares in an organisation that may in any way gain or lose financially from the publication of this manuscript, either now or in the future?
- Do you hold or are you currently applying for any patents relating to the content of the manuscript?
- Have you received reimbursements, fees, funding, or salary from an organization that holds or has applied for patents relating to the content of the manuscript?
- Do you have any other financial competing interests?
- Do you have any non-financial competing interests in relation to this paper?

If you can answer no to all of the above, write 'I declare that I have no competing interests' below. If your reply is yes to any, please give details below.

I declare that I have no competing interests

I agree to the open peer review policy of the journal. I understand that my name will be included on my report to the authors and, if the manuscript is accepted for publication, my named report including any attachments I upload will be posted on the website along with the authors' responses. I agree for my report to be made available under an Open Access Creative Commons CC-BY license (http://creativecommons.org/licenses/by/4.0/). I understand that any comments which I do not wish to be included in my named report can be included as confidential comments to the editors, which will not be published.

Choose an item.

To further support our reviewers, we have joined with Publons, where you can gain additional credit to further highlight your hard work (see: https://publons.com/journal/530/gigascience). On publication of this paper, your review will be automatically added to Publons, you can then choose whether or not to claim your Publons credit. I understand this statement.

Yes Choose an item.
