# Supplementary material for: BIGwas: Single-command quality control and association testing for multi-cohort and biobank-scale GWAS/PheWAS data
Source: Gigascience. 2021 Jun 29;10(6):giab047. doi: 10.1093/gigascience/giab047 (PMC8239664; doi:10.1093/gigascience/giab047)
Supplement: giab047_Reviewer_1_Report_Revision_1 — Shing Wan Choi -- 4/20/2021 Reviewed [file giab047_reviewer_1_report_revision_1.pdf]

Reviewer Report

Title: BIGwas - Single-command Quality Control and Association Testing for multi-cohort and biobank-scale GWAS/PheWAS data

Version: Revision 1 **Date:** 4/20/2021

Reviewer name: Shing Wan Choi

Reviewer Comments to Author:

In this update, the authors have addressed most of my concerns. In addition, the update to the README on their github sufficiently extensive, and I can now run the example data set after some minor changes. The paper should now ready to be published.

Some minor comment regarding the paper and the script:

1. Under "Assignment of variants to Pseudo-autosomal Regions", correct styling of "Plink" should be "PLINK"
2. The `qc-assoc-assets.sif` container is unsigned. As a result, singularity pull will fail and require user to pull the singularity image themselves. Users will also need to set `singularity.cacheDir` variable in the ` ~/.nextflow/assets/ikmb/gwas-qc/nextflow.config` file. Not sure why setting this variable in the local nextflow.config doesn't work.
3. label 'phase' for multiple processes were not defined, causing pipeline to fail when using with HPC scheduler. It seems like `phase` should use at least 8 CPUs (required by flashpca2_pruned_1kG process), but the default is 1.

Some suggestion that should:

- Considering that the HWE took up the most computation time, then a possibly quicker alternative might be the following:

1. Run `--hardy` once in each cohort independently (or maybe --frqx for quantitative traits).
2. Extract the GENO column from the .hwe file to obtain the A1 hom, het and A2 hom count for the control samples
3. For each SNP, you should now be able to calculate the number of A1 hom, het and A2 hom count of control samples with all possible cohort combinations.
4. Use the standalone hwe p-value calculation software here: https://github.com/chrchang/stats to calculate the p-value for each SNP and cohort combination

- The benefit of this approach is that the calculation of the A1, A2 and het count for each cohort combination is now reduced to a single summation and the p-value should be able to be calculated rather quickly. Hopefully this can help to speed up the current software

Level of Interest

Please indicate how interesting you found the manuscript: Choose an item.

Quality of Written English

Please indicate the quality of language in the manuscript: Choose an item.

Declaration of Competing Interests

Please complete a declaration of competing interests, considering the following questions:

- Have you in the past five years received reimbursements, fees, funding, or salary from an organisation that may in any way gain or lose financially from the publication of this manuscript, either now or in the future?
- Do you hold any stocks or shares in an organisation that may in any way gain or lose financially from the publication of this manuscript, either now or in the future?
- Do you hold or are you currently applying for any patents relating to the content of the manuscript?
- Have you received reimbursements, fees, funding, or salary from an organization that holds or has applied for patents relating to the content of the manuscript?
- Do you have any other financial competing interests?
- Do you have any non-financial competing interests in relation to this paper?

If you can answer no to all of the above, write 'I declare that I have no competing interests' below. If your reply is yes to any, please give details below.

I declare that I have no competing interests

I agree to the open peer review policy of the journal. I understand that my name will be included on my report to the authors and, if the manuscript is accepted for publication, my named report including any attachments I upload will be posted on the website along with the authors' responses. I agree for my report to be made available under an Open Access Creative Commons CC-BY license (http://creativecommons.org/licenses/by/4.0/). I understand that any comments which I do not wish to be included in my named report can be included as confidential comments to the editors, which will not be published.

Choose an item.

To further support our reviewers, we have joined with Publons, where you can gain additional credit to further highlight your hard work (see: https://publons.com/journal/530/gigascience). On publication of this paper, your review will be automatically added to Publons, you can then choose whether or not to claim your Publons credit. I understand this statement.

Yes Choose an item.
