# Supplementary material for: BIGwas: Single-command quality control and association testing for multi-cohort and biobank-scale GWAS/PheWAS data
Source: Gigascience. 2021 Jun 29;10(6):giab047. doi: 10.1093/gigascience/giab047 (PMC8239664; doi:10.1093/gigascience/giab047)
Supplement: giab047_Reviewer_2_Report_Original_Submission — Cristian Pattaro -- 1/2/2021 Reviewed [file giab047_reviewer_2_report_original_submission.pdf]

Reviewer Report

Title: BIGwas - Single-command Quality Control and Association Testing for multi-cohort and biobank-scale GWAS/PheWAS data

Version: Original Submission **Date:** 1/2/2021

Reviewer name: Cristian Pattaro

Reviewer Comments to Author:

Drs. KÃ¤ssens and Ellinghaus present BIGwas, a single-command analysis tool to jointly run data quality control and association analysis in the context of genome-wide association studies (GWAS). The Authors should be commended for dedicating time and releasing such a software, as there is great need of standardized quality control procedures in the GWAS field. I would like to point the authors' attention towards some points that, in my opinion, deserve some clarification.

- When I started reading the manuscript it took me a while before I realised that this is a tool for quality control from the individual data of each participant. Perhaps I was misled by the fact that many GWAS quality control softwares only focus on post-GWAS results. Perhaps this deserves immediate clarification at the beginning of the paper.
- Linked to the previous discussion, I would ask the authors to put the use of BIGwas a bit better into the context of consortia that include hundreds of studies, some very large like UKBB, others very small (1000-2000 samples): is there a real advantage to using BIGwas, given that many studies have their pipelines already implemented and many are not willing to change them? What might be the advantages/disadvantages?
- Since BIGwas is implemented in the context of GWAS consortia, in my opinion it would be appropriate not only to compare it with H3Agwas but also with mixed approaches (e.g. study-specific pipelines for post-GWAS analysis and quality control, eg GWAtoolbox or similar). If a formal comparison is not feasible, perhaps one could at least discuss advantages/disadvantages of alternative approaches.
- It seems that a limitation of BIGwas is the very strong reference to the IBDGC and Severe Covid-19 GWAS group consortia's protocols. This affects many aspects of the proposed analysis plan. For example, it is very common to find groups that do not use PLINK at all, neither at the quality control level nor at the analysis level; how should such groups behave in the management of e.g. the X chromosome or other steps if they wanted to use BIGwas?
- Apparently, the pipeline is thought for binary trait analysis (cases and controls). Is it also suitable for quantitative traits? Can the Authors specify this better?
- It is unclear whether the IBD QC step will exclude related individuals or if related individuals can be retained in the analysis by using linear mixed modeling approaches. These models could also incorporate a random batch effect, for instance, thus making the batch effect QC step unnecessary.
- In addition, to PLINK and SAIGE, there are other popular GWAS software such as BOLT-LMM, REGENIE, and others. Can they be implemented within the BIGwas analysis pipeline?
- GWAS and PheWAS are discussed under the same umbrella; however, the whole pipeline is presented and discussed only with regard to GWAS; please outline key differences between the two approaches or

limit to GWAS
- Please, explain all jargon. For instance, most audience might be unfamiliar with terms such as "containerized".
- In the Abstract, I would suggest to keep the approach general, avoiding referring to specific situations that might soon become outdated (eg: "the number of GWAS samples is twice as large as the current GWAS data release of the UK Biobank".
- Please, review text for typos. For instance, I found "per centage" in place of "percentage".

Level of Interest

Please indicate how interesting you found the manuscript: Choose an item.

Quality of Written English

Please indicate the quality of language in the manuscript: Choose an item.

Declaration of Competing Interests

Please complete a declaration of competing interests, considering the following questions:

- Have you in the past five years received reimbursements, fees, funding, or salary from an organisation that may in any way gain or lose financially from the publication of this manuscript, either now or in the future?
- Do you hold any stocks or shares in an organisation that may in any way gain or lose financially from the publication of this manuscript, either now or in the future?
- Do you hold or are you currently applying for any patents relating to the content of the manuscript?
- Have you received reimbursements, fees, funding, or salary from an organization that holds or has applied for patents relating to the content of the manuscript?
- Do you have any other financial competing interests?
- Do you have any non-financial competing interests in relation to this paper?

If you can answer no to all of the above, write 'I declare that I have no competing interests' below. If your reply is yes to any, please give details below.

I declare that I have no competing interests.

I agree to the open peer review policy of the journal. I understand that my name will be included on my report to the authors and, if the manuscript is accepted for publication, my named report including any attachments I upload will be posted on the website along with the authors' responses. I agree for my report to be made available under an Open Access Creative Commons CC-BY license (http://creativecommons.org/licenses/by/4.0/). I understand that any comments which I do not wish to

be included in my named report can be included as confidential comments to the editors, which will not be published.

Choose an item.

To further support our reviewers, we have joined with Publons, where you can gain additional credit to further highlight your hard work (see: https://publons.com/journal/530/gigascience). On publication of this paper, your review will be automatically added to Publons, you can then choose whether or not to claim your Publons credit. I understand this statement.

Yes Choose an item.
